# Bioenergetic and Metabolic Impairments in Induced Pluripotent Stem Cell-Derived Cardiomyocytes Generated from Duchenne Muscular Dystrophy Patients

**DOI:** 10.3390/ijms23179808

**Published:** 2022-08-29

**Authors:** Lubna Willi, Ifat Abramovich, Jonatan Fernandez-Garcia, Bella Agranovich, Margarita Shulman, Helena Milman, Polina Baskin, Binyamin Eisen, Daniel E. Michele, Michael Arad, Ofer Binah, Eyal Gottlieb

**Affiliations:** 1 Department of Physiology, Biophysics and Systems Biology, Rappaport Faculty of Medicine and Research Institute, Technion, Haifa 31096, Israel; 2 Department of Cell Biology and Cancer Science, Rappaport Faculty of Medicine and Research Institute, Technion, Haifa 31096, Israel; 3 Department of Molecular and Integrative Physiology, University of Michigan, Ann Arbor, MI 48109, USA; 4 Leviev Heart Center, Sheba Medical Center, Ramat Gan 52621, Israel; 5 Sackler Faculty of Medicine, Tel Aviv University, Tel Aviv 69978, Israel

**Keywords:** DMD, iPSC-CMs, bioenergetics, metabolism, electrophysiology

## Abstract

Duchenne muscular dystrophy (DMD) is caused by mutations in the *dystrophin* gene and dilated cardiomyopathy (DCM) is a major cause of morbidity and mortality in DMD patients. We tested the hypothesis that DCM is caused by metabolic impairments by employing induced pluripotent stem cell-derived cardiomyocytes (iPSC-CMs) generated from four DMD patients; an adult male, an adult female, a 7-year-old (7y) male and a 13-year-old (13y) male, all compared to two healthy volunteers. To test the hypothesis, we measured the bioenergetics, metabolomics, electrophysiology, mitochondrial morphology and mitochondrial activity of CMs, using respirometry, LC–MS, patch clamp, electron microscopy (EM) and confocal microscopy methods. We found that: (1) adult DMD CMs exhibited impaired energy metabolism and abnormal mitochondrial structure and function. (2) The 7y CMs demonstrated arrhythmia-free spontaneous firing along with “healthy-like” metabolic status, normal mitochondrial morphology and activity. In contrast, the 13y CMs were mildly arrhythmogenic and showed adult DMD-like bioenergetics deficiencies. (3) In DMD adult CMs, mitochondrial activities were attenuated by 45–48%, whereas the 7y CM activity was similar to that of healthy CMs. (4) In DMD CMs, but not in 7y CMs, there was a 75% decrease in the mitochondrial ATP production rate compared to healthy iPSC-CMs. In summary, DMD iPSC-CMs exhibit bioenergetic and metabolic impairments that are associated with rhythm disturbances corresponding to the patient’s phenotype, thereby constituting novel targets for alleviating cardiomyopathy in DMD patients.

## 1. Introduction

Duchenne muscular dystrophy (DMD), the most common of nine types of muscular dystrophy, is an X-linked disease affecting boys, teenagers and, rarely, adult heterozygous females. The incidence in male newborns is 1:3500 and the prevalence is 6:100,000 in the male population [1]. DMD, which is the most severe childhood form of dystrophy in the broader family of muscular dystrophies, is caused by mutations in the dystrophin gene encoding the dystrophin protein [2,3,4]. At the early stages of the disease, DMD affects the shoulders, upper arms, hips and thigh muscles, resulting in weaknesses that lead to difficulties in maintaining balance. As age advances, DMD is associated with impairments in both pulmonary and cardiac functions, such as respiratory failure, and clinical symptoms of cardiomyopathy ranging from mild cardiac dysfunction to severe dilated cardiomyopathy (DCM) [5]. While improvements in respiratory therapy have extended patient lifespan, more patients have died from cardiomyopathy in the past 20 years, with a current rate of >30% of DMD patients dying from DCM [6,7,8,9]. The severity of DMD cardiomyopathy, which commonly appears at the ages of 6 to 18-year-old, increases with age. Hence, >90% of 18-year-old male DMD patients demonstrate evidence of cardiac dysfunction [10]. At the final stages of the disease, severe heart failure increasingly becomes the leading cause of death [6,7,10,11].

Dystrophin is a key structural/functional protein that provides strength and stability to the contracting muscle and is essential for maintaining healthy muscle function. A lack of functional dystrophin leads to cell damage, impaired Ca^2+^ homeostasis, elevated oxidative stress and decreased energy production in muscle cells. Dystrophin deficiency in DMD results in an unstable muscle fiber structure and, therefore, the contracting cells are adversely affected by continuous contractions [10,12,13]. Consequently, loss of sarcolemmal dystrophin and dystrophin–glycoprotein complex (DGC) promotes muscle fiber damage during muscle contraction [14]. Although dystrophin is essentially a structural protein, studies in DMD patients and in the DMD mouse model *mdx* [15,16] showed that dystrophin mutations are associated with metabolic changes that may contribute to functional aberrations, including electrophysiological abnormalities and arrhythmias [17,18]. Specifically, Hughes and co-workers [15] compared mitochondrial bioenergetics with functional and histopathological indices of myopathy in damaged muscles early in DMD (4 weeks) in D2.B10-DMD^mdx^/2J mice (D2.*mdx*). These authors reported that Complex I-supported maximal H_2_O_2_ emission was elevated, and that ADP had a reduced ability to attenuate emission during respiration. This was associated with an impaired ability of ADP to stimulate respiration at sub-maximal and maximal kinetics, as well as a loss of creatine-dependent mitochondrial phosphate shuttling in the diaphragm and quadriceps. Hence, while a common paradigm is that excessive Ca^2+^ influx into affected myofibers is one of the major initiating causes for dystrophinopathy, emerging evidence suggests that metabolic and mitochondrial dysfunction play significant roles in disease progression [13,19,20,21,22,23,24,25,26]. For example, Lindsay et al., reported that global metabolic impairment is associated with *mdx* disease progression and that tricarboxylic acid (TCA) cycle deficiencies are a downstream consequence of dystrophin loss. Due to the major DMD-related deficits in mitochondrial function, which impact on intracellular Ca^2+^ levels and metabolic balance, it has been proposed that DMD is primarily a mitochondrial myopathy [13,27]. These impairments reduce energy production capacity [19] and resting energy levels [28], thereby severely limiting the muscle’s ability to reduce damage and facilitate muscle repair. Accordingly, in this study we investigated the hypothesis that *dystrophin* mutations in DMD lead to cardiomyopathy-causing bioenergetic/metabolic impairments, which could be therapeutically targeted to improve cardiac function.

## 2. Results

### 2.1. Metabolic Profiling Revealed a Bioenergetic Impairment of DMD iPSC-CMs

To obtain an overview of the metabolic status of DMD cardiomyocytes, we first devised an untargeted strategy using liquid chromatography–mass spectrometry (LC–MS)-based metabolomics focusing on polar metabolites (such as phopsho-sugars, organic acids, nucleotides and even fatty acids). This approach enabled us to discover and characterize critical changes/trends in different aspects of the central carbon metabolism of iPSC-CMs. The study included six iPSC-CM sources: two healthy controls (one male and one female) and four DMD patients. (1) A 32-year-old male DMD patient carrying a substitution of cytosine to thymine (c.5899C > T) constituting a premature stop codon. (2) A 50-year-old female DMD patient carrying a deletion of exons 8–12 (ex.8_12del). These two patients were included in our recent publication [29]. (3) A 13-year-old (13y) male DMD patient lacking exons 45–50 of the dystrophin gene. (4) A 7-year-old (7y) male DMD patient carrying a nonsense mutation (c.8038C > T) that led to premature termination of translation and a truncated dystrophin protein.

**Figure 1:**
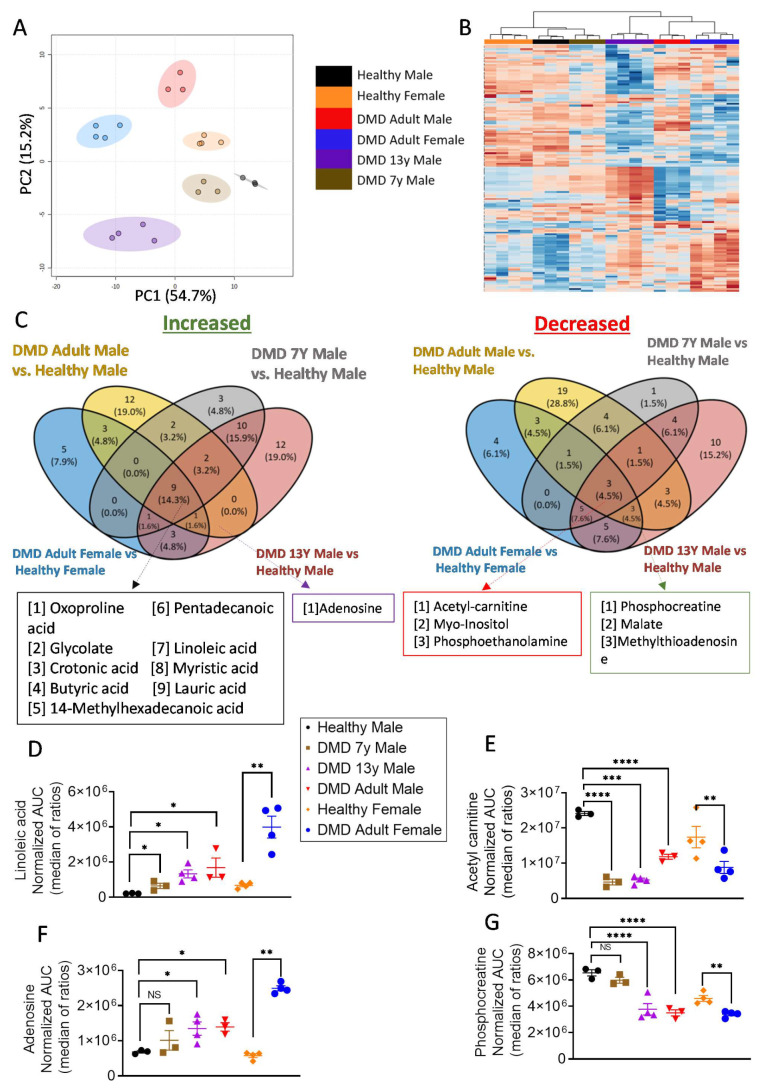
Metabolomics analysis of iPSC-CMs. (**A**) PCA of 1700 chromatographic feature intensities for iPSC-CMs, colored by group: DMD adult male (black, n = 3), DMD adult female (blue, n = 4), DMD 13-year-old (13y) male (purple, n = 4), DMD 7-year-old (7y) male vs. healthy male (brown, n = 3) and healthy female (orange, n = 3). The PCA plot was created using Metaboanalyst. Analysis was applied on the log-transformed intensities, normalized by the median with auto-scaling. The colored ellipses represent 95CI areas. Group separation as captured by the first principal component distinguished more severe DMD phenotypes from the less severe and healthy ones. (**B**) Unsupervised clustering analysis presented by heatmap. Three of the four DMD CMs were clustered together apart from the healthy control, while the arrhythmia-free 7y CMs were clustered with the healthy control. The heatmap was created in Metaboanalyst, using the same chromatographic feature intensities as in panel (**A**) (Ward clustering, Euclidean distance). (**C**) Venn diagrams showing that the intersections of metabolites either increased (**left**) or decreased (**right**) in the comparisons indicated in the labels (DMD adult male vs. healthy male, DMD 13y male vs. healthy male, DMD 7y male vs. healthy male and DMD adult female vs. healthy female). The metabolites present in most relevant intersections are listed below in the colored boxes. (**D**,**E**) Linoleic acid (**left**) as a representative of fatty acid accumulation in all DMD samples coupled with a decrease in acetyl carnitine (**right**). (**F**,**G**) An increase in adenosine coupled with a decrease in phosphocreatine in three out of four DMD samples as an indication of energy crisis. The Shapiro–Wilk test of normality was applied for all metabolite features to assess whether the data were normally distributed. Data were normalized to the median of the ratios. One-way ANOVA for male original samples followed by *post hoc* Tukey’s test, two-sided *t*-test for female original samples. * *p* < 0.05, ** *p* < 0.01, *** *p* < 0.001, **** *p* < 0.0001.

**Figure 2:**
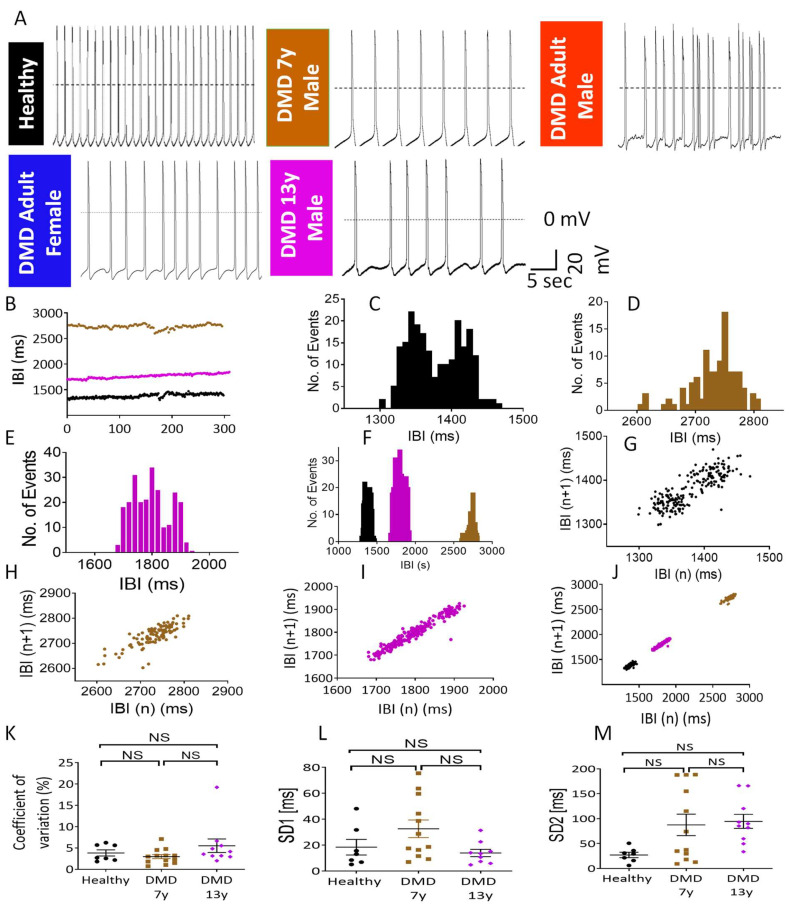
Electrophysiological features and action potential characteristics of spontaneously firing healthy and DMD iPSC-CMs. (**A**) Representative action potentials recorded from iPSC-CMs generated from the healthy control (firing regularly), DMD 7year old male (7y) (firing regularly) and the DMD adult male, adult female and 13 year old (13y) male, all displaying arrhythmogenic firing patterns. (**B**–**M**) Beat rate variability (BRV) analysis of spontaneously firing CMs from the 5 experimental groups. (**B**) Superimposed representative inter-beat interval (IBI) vs. time plots of healthy (black symbols), DMD 7y (brown symbols) and DMD 13y (purple symbols) iPSC-CMs. (**C**–**E**) Representative IBI histograms for the experiments shown in (**B**). (**F**) Superimposed IBI histograms for the three groups. Poincaré plots of healthy (**G**), DMD 7y (**H**) and DMD 13y (**I**) CMs. (**J**) Superimposed Poincaré plots for the three groups. (**K**) Coefficient of variation analysis. (**L**) Standard deviation 1 (SD1) analysis. (**M**) SD2 analysis. Healthy, n = 7; DMD 7y, n = 13; DMD 13y, n = 10. Kruskal–Wallis test for non-normal distribution; NS = non-significant.

Using LC–MS-based metabolomics, we detected an average of ~1700 MS features (i.e., signal peaks in each chromatographic retention period of the analysis) using Compound Discoverer™ software (Compound Discoverer 3.2, Thermo Fisher Scientific, Carlsbad, CA, USA). The intensities of these features correlated with their concentration in the sample, thus allowing a relative comparison between DMD and healthy control iPSC-CMs. Interestingly, the overall metabolic variability captured by the unsupervised principal component analyses (PCA) and heatmap clustered three (adult male, adult female and 13y) of the four DMD iPSC-CMs away from the healthy control (Figure 1), while the arrythmia-free (Figure 2) 7y male-derived iPSC-CMs was clustered with the normal control (Figure 1A,B). Therefore, although unsupervised untargeted metabolomics did not provide a complete understanding of the changes in specific metabolic traits as a stand-alone method, it strongly indicated that aberrant metabolism is an integral part of the phenotypic consequences of *dystrophin* mutations in DMD.

To identify specific metabolic profiles, we performed targeted analyses using an in-house database of metabolite standards. Consequently, we were able to identify signatures for ~120 key metabolites of the central carbon metabolism and obtained their respective high-quality relative intensity values. The metabolic signatures of all DMD patient-derived CMs, compared to their relevant gender controls, are depicted in the waterfall plots in Supplementary Figure S2A–D. Overall, nine metabolites were significantly higher and three metabolites were significantly lower in all DMD CMs compared to control (Figure 1C). These common metabolic alterations suggest an impairment in fatty acid oxidation in DMD CMs, since several fatty acids were highly present in these cells (particularly linoleic acid, which is the major fatty acid supplemented to these cells), while an accompanied decrease in acetyl-carnitine, an intermediate metabolite of fatty acid oxidation, was observed (Figure 1C–E and Supplementary Figure S2). However, when searching for the unique metabolic signatures of the CMs derived from adult and 13y DMD patients, an increase in adenosine and a decrease in phosphocreatine (PCr) were detected (Figure 1C,F,G). These additional metabolic alterations are indicative of an energy imbalance in the affected CMs. Adenosine accumulation is derived from ATP/ADP/AMP degradation, while PCr serves as a rapid labile reserve of high-energy phosphates in the myocardium (and skeletal muscles) used to recycle ATP; therefore low PCr levels imply a severe deficit in energy metabolism (Supplementary Figure S3). It is worth mentioning that PCr was also lower in the healthy female control in comparison to the healthy male (Figure 1G). In general, there are differences between males’ and females’ metabolic profiles, including in iPSC-CMs [30]. Interestingly, the enzyme creatine kinase is higher in males than in females [31]; hence, one may expect that PCr would be higher in male samples in general.

### 2.2. The Metabolic/Energy Status of the iPSC-CMs Correlates with Their Electrophysiological Characteristics

To test the hypothesis that the metabolic phenotypes of mutated iPSC-CMs reflect not only the genotype (a spectrum of *dystrophin* mutations) but also the clinical status of the patients, we compared the electrophysiological properties of the metabolically characterized CMs. We previously reported that adult male and female iPSC-CMs (derived from clinically DMD-DCM patients) demonstrated electrophysiological deficits, including arrhythmias [21] (Figure 2A). CMs derived from the DMD 7y and DMD 13y patients were analyzed to determine whether their electrophysiological characteristics were in accordance with their identified energetic states and the overall disease status of these young patients. Indeed, in agreement with their (healthy-like) energetic status, the 7y CMs fired regularly (albeit at a slower rate), similarly to healthy CMs (Figure 2A) and in contrast to the arrhythmogenic behavior of the DMD adult male [29], the DMD adult female [29] and the DMD 13y CMs (Figure 2A). Specifically, in the DMD 7y, none of the 11 CMs analyzed were arrhythmogenic, whereas in the DMD 13y, 9 of the 20 CMs analyzed presented arrhythmias. These findings show that despite the presence of the *dystrophin* mutation in the DMD 7y CMs, the firing pattern was regular. The DMD 7y CMs resembled healthy CMs, not only in the lack of arrhythmias but also in the following action potential characteristics: dV/dt_max_, action potential amplitude (APA) and maximal diastolic potential (MDP) (Supplementary Figure S4). Despite these similarities, action potential duration (APD) at 20% and 90% repolarization (APD_20_/APD_90_) (Supplementary Figure S4) was prolonged in the DMD 7y CMs. That this prolongation was not due to the inherent lower firing rate was demonstrated by the prolonged (compared to healthy) Bazett’s-corrected APD_90_ (cAPD_90_-B), Hodge’s-corrected APD_90_ (cAPD_90_-H) and Fridericia’s-corrected APD_90_ (cAPD_90_-Fri) (Supplementary Figure S4). Importantly, these results demonstrate that although the DMD 7y arrhythmia-free CMs presented healthy-like bioenergetic/metabolic status, the slow spontaneous firing rate and prolonged APD (compared to healthy CMs) indicated that electrophysiological deficits were already beginning to emerge in these young CMs. As for the DMD 13y CMs, the spontaneous firing rate was similar to the DMD 7y CMs, both being slower than healthy CMs. The APA, MDP and dV/dt_max_ were comparable in healthy, DMD 7y and 13y CMs. Further, APD_20_, cAPD_90_-B and cAPD_90_-Fri were longer in the 13y than in the healthy CMs and in general similar to the APD prolongation in the 7y CMs.

To compare the dynamic patterns of spontaneously firing DMD 7y and 13y vs. healthy CMs, we analyzed the beat rate variability (BRV) using tools employed in our previous studies [29,32,33]. In agreement with the absence of arrhythmias in healthy and 7y CMs, the inter-beat interval (IBI) vs. time plots (Figure 2B) and the IBI histograms (Figure 2C–F) illustrated low dispersions of intervals, suggesting homogenous cardiomyocyte populations firing regularly. This regularity was further demonstrated by the cigar-like Poincaré plot clouds with coefficients of variation (CV), SD1 and SD2 (Figure 2K–M) similar to healthy CMs. Importantly, although the 13y CMs presented arrhythmias (which are not an all-or-none phenomenon), their magnitude was mild compared to DMD adult CMs [29], thereby resulting in BRV indices similar to healthy CMs (Figure 2K–M). In contrast, adult DMD CMs presented prominent arrhythmias and, hence their BRV indices were significantly different from those of healthy CMs [29].

### 2.3. Stable-Isotope Glucose Tracing Reveals Decreased Oxidative Status in DMD Cardiomyocytes

**Figure 3:**
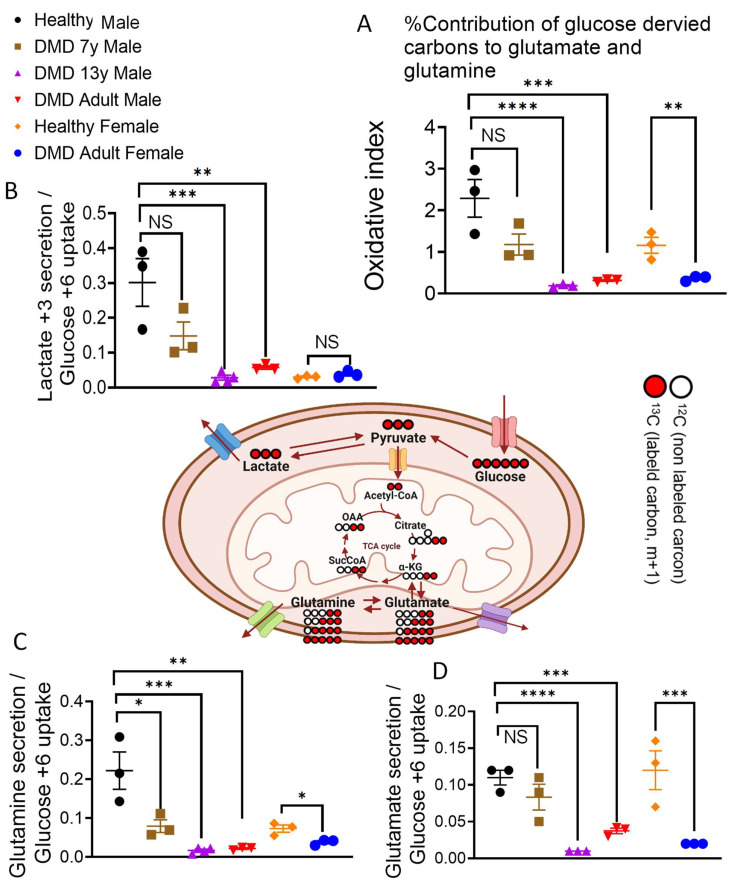
Glucose tracing in DMD iPSC-CMs. LC-MS measurements of labeling pattern for glycolytic and TCA cycle derivatives indicated as lactate (**B**), glutamate (**D**), and glutamine (**C**) secretion following 24 hours incubation with ^13^C_6_-labeled glucose. Labeling patterns of glucose derived carbons are detailed in the middle scheme. DMD adult male, adult female and the 13-year-old (13y) CMs showed a decrease in oxidative metabolism compared to healthy and the DMD 7-year-old (7y) CMs. Oxidative index (**A**) is calculated as the ratio between the weighted sum of ^13^C-glucose-derived secreted glutamate (**B**) and glutamine (**C**) to the uptake of ^13^C_6_-glucose. The Shapiro–Wilk test of normality was applied for all metabolites to assess whether the data were normally distributed. Two tailed homoscedastic *t*-test over log-transformed data; NS = non-significant, * *p* < 0.05, ** *p* < 0.01, *** *p* < 0.001, **** *p* < 0.0001.

Our results thus far imply that, while DMD CMs may demonstrate alterations in fatty acid oxidation, cells that overcome the energetic crisis (i.e., DMD 7y CMs), potentially via the utilization of other oxidizable substrates (Supplementary Figure S3), would not develop arrhythmias. Due to the apparent faulty energetic status of the DMD CMs, we performed a ^13^C_6_-labeled glucose tracing experiment designed to study the potential deficiencies in energy metabolism. Stable isotope tracing enables the detection of metabolites derived from the labeled metabolic tracer, which, in the present study, was ^13^C_6_-glucose. Each ^13^C_6_-glucose-derived carbon incorporated into any metabolite resulted in a mass shift of ~1Da; hence, the cellular fate of glucose could be extensively studied using LC-MS. Accordingly, CMs from all DMD patients were incubated for 24 h in the presence of ^13^C_6_-glucose before metabolite extraction. We investigated the relative changes in the different glucose-derived metabolites in the culture media in order to infer the rate of production of glucose-derived metabolites from glycolysis and the TCA cycle (Figure 3).

Using glucose tracing, we discovered that in adult and DMD 13y CMs, there was a diminished entry of glucose-derived carbon into the TCA cycle, which was manifested as attenuated secretion of glucose-derived ^13^C isotopes in glutamate and glutamine (Figure 3). This was indicative of decreased glucose oxidation in the mitochondria. To better characterize the oxidative state of the CMs, we used an “oxidative index” defined as the weighted ratio of secreted ^13^C-labeled glutamate and glutamine normalized to the ^13^C-glucose uptake (Figure 3). While the DMD 7y CMs showed a somewhat diminished glucose oxidation compared to control, the oxidative indexes of the arrhythmogenic DMD 13y CMs were significantly lower.

### 2.4. DMD-Derived iPSC-CMs Demonstrate Oxidative Phosphorylation Deficiency

**Figure 4:**
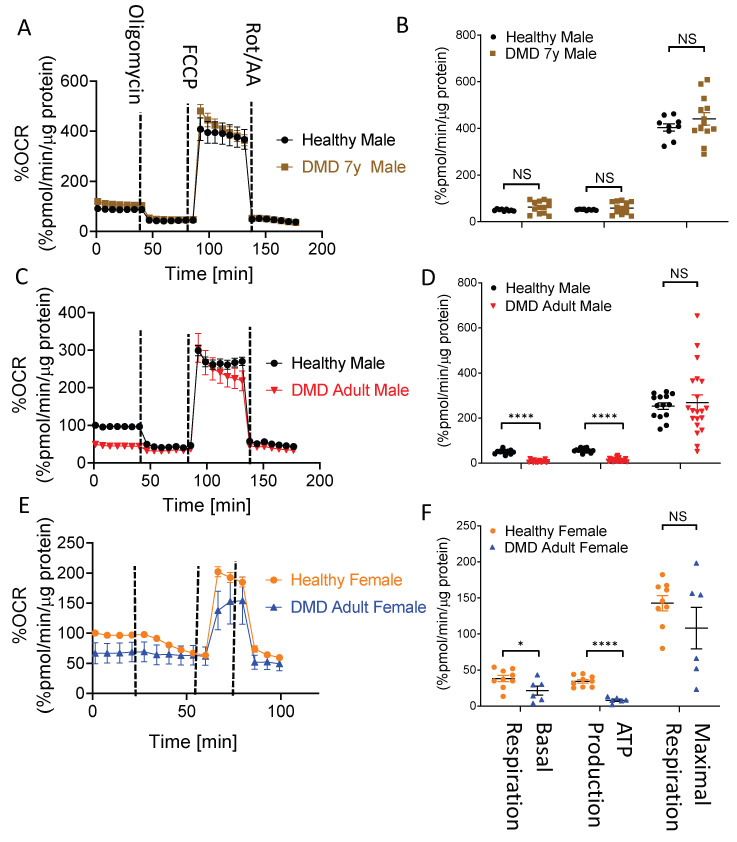
Respiration profiles for DMD vs. healthy control iPSC-CMs. (**A**,**C**,**E**) Oxygen consumption rate (OCR) in DMD 7-year-old (7y), DMD adult male and DMD adult female compared to healthy CMs. (**B**,**D**,**F**) Basal respiration, ATP production and maximal respiration rates in the different experimental groups. OCR was recorded during sequential additions of respiration modulators: (1) oligomycin, (2) FCCP and (3) antimycin A and rotenone. Basal respiration, ATP production and maximal respiration rates calculated according to Supplementary S5 and based on the relevant OCR plots for each cell line. OCR values are normalized to μg protein measured using the modified Lowry protein assay and are compared to the basal OCR of a health (defined as 100%). Healthy, n = 9–14; DMD 7y, n = 13; DMD adult male, n = 20; DMD adult female, n = 6. Kruskal–Wallis test (for non-normal distribution); NS = non-significant, * *p* < 0.05, **** *p* < 0.0001.

To test the hypothesis that *dystrophin* mutations are associated with bioenergetic/metabolic aberrations in general, and oxidative metabolism in particular, we employed the Seahorse XFe96 metabolic flux analyzer to measure the two major energy-producing pathways—mitochondrial respiration and glycolysis. The status of the mitochondrial oxidative phosphorylation pathway was represented by the oxygen consumption rate (OCR) (Supplementary Figure S5) under basal conditions and following the addition of: (1) oligomycin, an ATP synthase inhibitor that decreases OCR and enables the calculation of ATP-producing OCR from the basal respiration rate; (2) FCCP, an ionophore that uncouples oxygen consumption from ATP production by dissipating the mitochondrial proton gradient, thus enabling the measurement of the maximum respiratory capacity; (3) a mixture of rotenone and antimycin A to inhibit complexes I and III of the mitochondrial respiratory chain, respectively. Accordingly, we performed the test with healthy male, healthy female, DMD 7y, DMD adult male and DMD adult female CMs (Figure 4). While the respiration profile for healthy CMs was comparable to DMD 7y CMs (Figure 4A,B), in DMD adult male and female CMs, there were significant decreases of 80% and 45% in basal respiration, respectively, and a significant decrease of 75% in ATP production coupled to oxygen consumption (Figure 4C–F). These findings demonstrate that, while DMD adult male and female CMs exhibited impaired bioenergetic status, the DMD 7y CMs’ bioenergetic features were similar to those of healthy CMs. Interestingly, in the affected adult DMD CMs, the respiration rate was restored following the uncoupling of ATP synthesis from the proton gradient generated by the electron transfer respiratory chain (Figure 4C–F; following FCCP addition). This suggests that the major defect of the mitochondria is in the ATP synthase (Complex V). It is worth mentioning that no compensatory glycolytic induction, which would have been indicated by a lack of change in the extracellular acidification rate (ECAR) (Supplementary Figure S6) or lactate secretion (Figure 3), was seen in affected DMD CMs. Such deficits in oxidative phosphorylation unaccompanied by a compensatory increase in glycolysis can explain the energy crisis observed in these CMs (Figure 1F,G).

### 2.5. Mitochondria Structural and Functional Alterations in Energy-Depleted DMD iPSC-CMs

**Figure 5:**
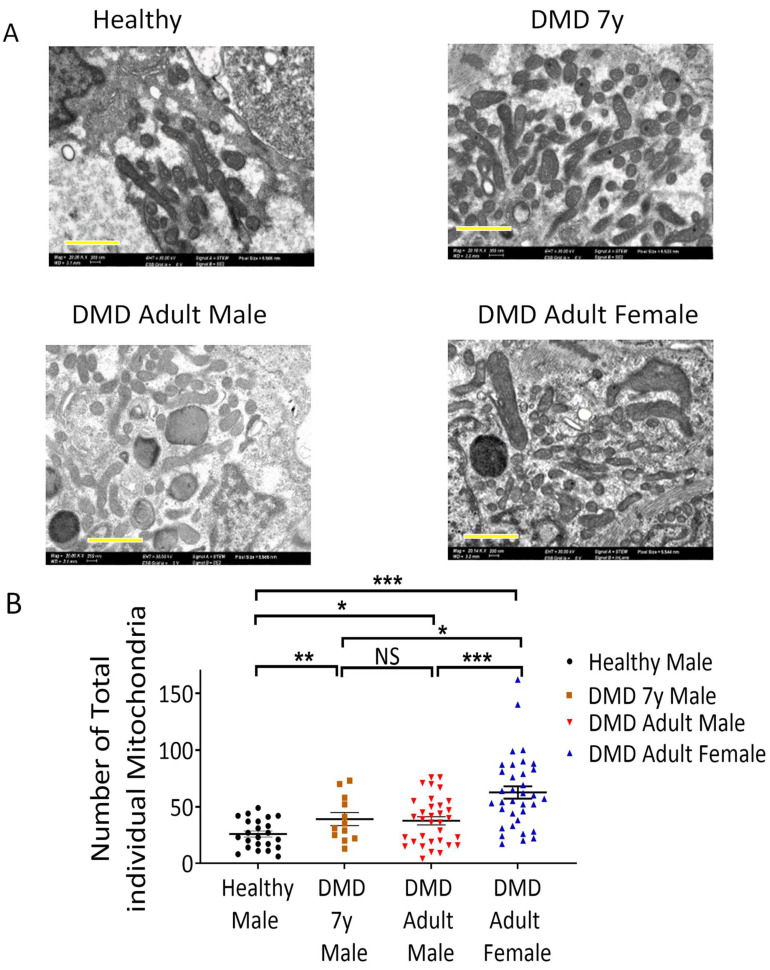
Electron microscopy analyses of mitochondria in iPSC-CMs. (**A**) Mitochondrial morphology and number in healthy control, DMD 7-year-old (7y), DMD adult male and DMD adult female CMs acquired by transmission electron microscopy. Scale bar 500 nm. (**B**) Quantification of the total number of individual mitochondria displayed a significant increase in mitochondria number in DMD 7y, DMD adult male and DMD adult female CMs. Mitochondria counted: healthy, n = 595; DMD 7y, n = 554; DMD adult male, n = 1359; DMD adult female, n = 21,555. Abnormal mitochondria: healthy, n = 11; DMD 7y, n = 11; DMD adult male, n = 296; DMD adult female, n = 556. The Anderson–Darling test of normality was applied for all metabolites to assess whether the data were normally distributed. One-way ANOVA, NS = non-significant, * *p* < 0.05, ** *p* < 0.01, *** *p* < 0.001.

**Figure 6:**
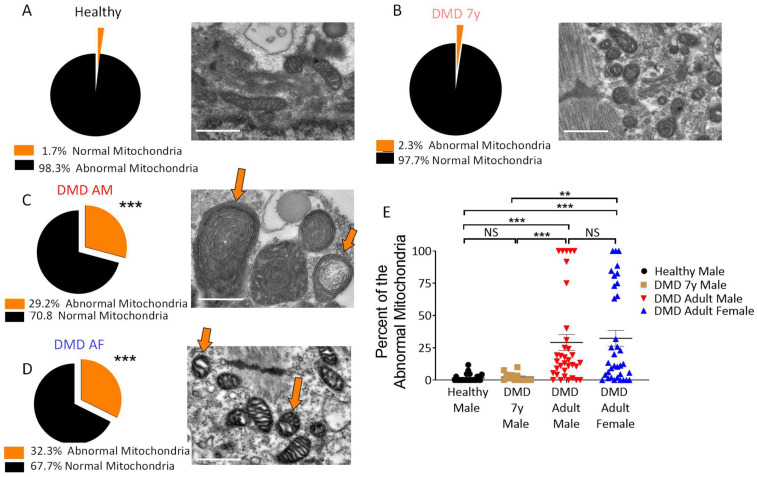
Morphological alterations in DMD mitochondria. Representative transmission electron microscopy (TEM) and analysis of healthy control (**A**), DMD 7-year-old (7y) (**B**), DMD adult male (AM) (**C**) and DMD adult female (AF) (**D**) CMs. The mitochondria abnormalities in DMD adult male and female CMs included increased size, reduced matrix density and disrupted cristae (orange arrows). (**E**) A summary of the abnormal mitochondria in the four experimental groups. The extent of abnormal mitochondria was calculated as the percentage of abnormal mitochondria among the total individual mitochondria (normal + abnormal mitochondria). The summary shows a significant increase in abnormal mitochondria percentage in DMD adult male and female CMs compared to DMD 7y and healthy control CMs. Normal mitochondria: healthy, n = 584; DMD 7y, n = 543; DMD adult male, n = 1063; DMD adult female, n = 1599. Abnormal mitochondria: healthy, n = 11; DMD 7y, n = 11; DMD adult male, n = 296; DMD adult female, n = 556. Welch’s ANOVA test (for non-parametric distribution) was used for statistical comparisons. ** *p* < 0.01, *** *p* < 0.001, NS = not significant. Scale bar 500 nm.

As energy conservation represented by PCr and ATP synthesis is managed by mitochondria, it is likely that the impaired energy metabolism in DMD CMs is associated with abnormal mitochondria structure and/or function [34,35]. To determine mitochondria number and morphology, electron microscopy (EM) analysis was performed in DMD adult male, adult female, 7y and healthy CMs. As illustrated in Figure 5, DMD adult male, adult female and 7y CMs contained more individual mitochondria than healthy CMs. Mitochondrial aberrations, including increased size, disrupted cristae and multiple focal swelling areas, were frequently seen in DMD adult CMs (indicated by orange arrows in Figure 6 and Supplementary Figure S7) but not in DMD 7y and healthy CMs. Specifically, 29% and 32% of mitochondria in DMD adult male and female CMs, respectively, presented abnormal morphologies, while only ~2% abnormal mitochondria were detected in both the DMD 7y and healthy CMs (*p* < 0.001) (Figure 6). These alterations of mitochondria morphology in DMD adult CMs are likely to be associated with abnormal oxidative phosphorylation, which impacts energy metabolism.

**Figure 7:**
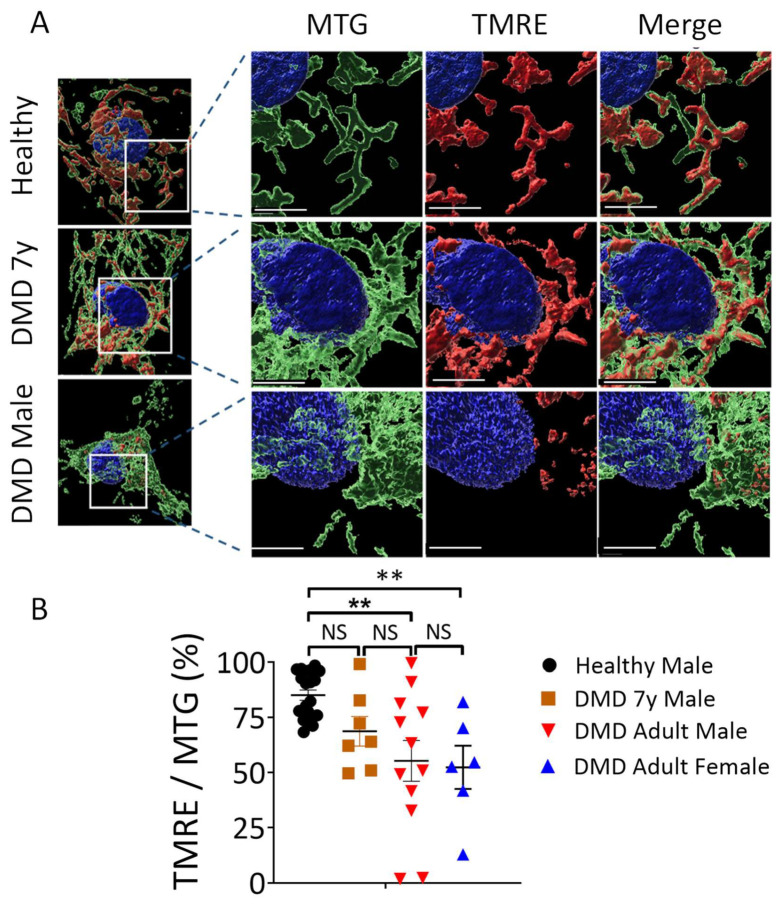
Mitochondrial activity in beating iPSC-CMs. (**A**) Representative images of mitochondria in healthy, DMD 7-year-old (7y), DMD adult male and adult female CMs simultaneously stained with three fluorescent dyes: MTG (green; ΔΨ independent), TMRE (red; ΔΨ dependent) and Hoechst (blue; nuclear staining for live cells). Scale bar, 8 μm. (**B**) Quantification of total mitochondrial activity calculated by the ratio of the volume of fluorescence (TMRE/MTG) for each Z-stack image. The summary shows a significant decrease in DMD adult male and female CMs compared to healthy and DMD 7y CMs. Healthy, n = 7; DMD 7y, n = 6; DMD adult male, n = 12; DMD adult female, n = 6. The Shapiro–Wilk test of normality was applied for all metabolites to assess whether the data were normally distributed; One-way ANOVA, NS = non-significant, ** *p* < 0.01.

To determine whether these morphological abnormalities were associated with impaired mitochondrial function, we measured the mitochondrial activity in beating CMs by acquiring live confocal 3D images. Mitochondrial activity was determined from the ratio of the volume of active mitochondria to the volume of total mitochondria. Specifically, we simultaneously used MitoTracker Green (MTG) and tetramethylrhodamine ethyl ester (TMRE) fluorescent staining to label total and active mitochondria, respectively; Hoechst dye was used for nuclear live staining (Figure 7). MTG accumulates in mitochondria independently of changes in mitochondrial membrane potential (ΔΨ_M_) while TMRE is sensitive to changes in ΔΨ_M_ (positively-charged red-orange dye) and accumulates specifically in active mitochondria. As a control, ΔΨ_M_ was depolarized with FCCP and, hence, the TMRE/MTG ratio was diminished (Supplementary Figure S7). Furthermore, incubating CMs with oligomycin which inhibits ATP synthase, resulted in a lack of TMRE staining (Supplementary Figure S8). This functional mitochondrial analysis revealed that in agreement with the metabolic and morphologic findings, whereas DMD adult male and female mitochondrial activity levels were attenuated by 45% and 48%, respectively, the DMD 7y mitochondria activity was comparable to healthy CMs.

## 3. Discussion

Our goal was to investigate the hypothesis that *dystrophin* mutations are associated with metabolic deficits causing cardiac dysfunction. The main findings were: (1) Adult DMD male and female CMs exhibited bioenergetic and metabolic deficiencies reflected in impairments in the PCr energy system, oxidative phosphorylation pathway, oxidative index, mitochondrial activity and morphology. (2) The DMD 7y CMs demonstrated arrhythmia-free spontaneous firing patterns associated with “healthy-like” bioenergetic metabolic status and normal mitochondrial morphology and function. In contrast, the 13y CMs were arrhythmogenic, but not as prominently so as DMD adult male and female CMs, and showed decreased PCr levels.

### 3.1. Decreased PCr in DMD CMs

A major finding was that PCr was markedly decreased in DMD adult (male and female) and 13y CMs but not in the arrhythmia-free DMD 7y CMs (Figure 1G). This may have been because PCr contains a high-energy phosphate—a necessary source for muscle contractions, it reflects the energy state of the muscle [36,37,38]. In support of the decreased PCr in DMD CMs, Pulido et al., reported [39] that PCr levels in *mdx* myotubes were about half of those in control mice. Insufficient metabolic energy was reported in *mdx* mice, especially under conditions of higher demand for ATP during exercise [19]. The association between *dystrophin* mutations, low PCr concentration and cardiac dysfunction was also reported in DMD patients and *mdx* mice [13,40]. Finally, ex vivo studies of *mdx* mice hearts similarly demonstrated decreased PCr levels combined with lower mitochondrial content [40].

### 3.2. Mitochondrial Dysfunction in DMD CMs

In agreement with the low energy status reflected by the PCr levels, DMD adult male and female CMs demonstrated a marked decrease in oxidative phosphorylation capabilities, indicated by the level of oxygen consumption coupled to ATP production (Figure 4). The oxidative phosphorylation capacity of CMs was assessed by the OCR before and after ATP synthase inhibition by oligomycin (Figure 4) and by the ensuing glucose fate in CMs from carbon tracing of ^13^C_6_-glucose to either glutamate and glutamine (oxidatively via the TCA cycle) or reductively to lactate (Figure 3). These results strongly support the notion that iPSC-CMs are a trustworthy model for the DMD disease, as manifested by mitochondrial dysfunction. In support of the attenuated oxidative phosphorylation capacity of DMD CMs, by acquiring live confocal 3D images of TMRE- and MTG-stained CMs, we found that adult male and female mitochondrial activity levels were attenuated by 45% and 48%, respectively, whereas the 7y mitochondria activity was comparable to healthy CMs. What follows are representative studies supporting our studies in DMD CMs. (i) Hughes et al., studied D2.B10-DMD*^mdx^*/2J mice and found that, at 4 weeks of age, prior to cardiac remodeling or cardiac dysfunction, there were impairments in ADP-stimulated respiration, in ADP attenuation of H_2_O_2_ emission and in the ability of creatine to enhance ADP’s control of mitochondrial bioenergetics [15]. (ii) Based on their studies in *mdx* mice, Moore et al., recently reported that, before the onset of myofiber necrosis, mitochondrial and metabolic abnormalities are present [41]. (iii) Kyrychenko et al., employing electrophysiological and imaging techniques, showed in cardiomyocytes from *mdx* mice that the mitochondrial matrix was progressively oxidized and that the number of damaged mitochondria gradually increased. Degradation in mitochondrial structure was correlated with a progressive increase in mitochondrial Ca^2+^ sequestration and mitochondrial depolarization, despite a substantial and persistent elevation in resting cytosolic sodium levels. A comprehensive review of the metabolic alterations in cardiomyocytes of patients with Duchenne and Becker muscular dystrophies was provided by Esposito and Carsana [16]. In summary, our novel findings derived from bioenergetics and respiration measurements, metabolomics, the mitochondrial structure and function of DMD iPSC-CMs and previous reports led to the important conclusion that *dystrophin* mutations are linked to attenuated mitochondrial oxidative phosphorylation.

### 3.3. Changes in Mitochondrial Content and Morphology in DMD CMs

In addition to the mitochondrial dysfunction represented by attenuated activity and oxidative phosphorylation capacity in DMD CMs, DMD CMs had higher mitochondrial content and increased numbers of mitochondria with abnormal morphologies. Whereas the presence of abnormal mitochondria is expected and will be discussed below, an unexpected finding was that DMD adult CMs and 7y CMs contained more individual mitochondria than healthy CMs, which may constitute a compensatory mechanism for defective bioenergetic/metabolic pathways. In agreement with our findings, Kang et al. [42] reported that electron micrographs of cardiac muscle sections revealed a significantly larger number of mitochondria with losses of normal cristae structure in *mdx* cardiac muscle compared to WT tissue. Moreover, the number of structurally abnormal mitochondria significantly increased in the cardiac tissue of 12 months and older dystrophic animals that ad developed dilated cardiomyopathy. In support of this report, Onopiuk et al. [43] reported that myoblasts derived from *mdx* mice exhibited disorganized mitochondrial networks. Further, using a *Caenorhabditis elegans* model for DMD, Giacomotto et al. [44] showed that the morphology of the mitochondrial network reflected by the GFP signal observed with confocal microscopy revealed dramatic mitochondrial fragmentation, which further increased with age. Similar mitochondrial fragmentation was also found in a zebrafish model for DMD. Finally, Dubinin et al. [45] reported that mitochondria from *mdx* mice were spherical structures with an irregular organization of mitochondrial cristae, and some mitochondria contained vacuoles and abnormalities in the outer membrane.

### 3.4. The Link between Dystrophin Mutation and Mitochondrial Structural and Functional Aberrations

A major issue not yet fully resolved is the link between *dystrophin* mutations and the specific observed mitochondrial abnormalities, including morphological and bioenergetic/metabolic aberrations. As noted by Hughes et al. [15], *dystrophin* mutations cause the disruption of sarcolemal stability and cytoskeletal organization, which results in several intracellular stressors contributing to disease progression and cardiomyopathy. Among these stressors are aberrations in intracellular calcium regulation (e.g., [46]) and oxidative stress, both of which can contribute to mitochondrial dysfunction. Hence, constant tearing of the sarcolemma was shown to cause an influx of extracellular calcium ions, thereby causing intracellular calcium overload. In this regard, the sacrcolemmal contribution to abnormal calcium handling has been comprehensively reviewed by Mareedu and co-workers [46] and Zablocka et al. [47]. Briefly, high intracellular calcium can cause several damaging downstream cascades, affecting the mitochondria. Under abnormally high intracellular calcium concentrations, the calcium overload in mitochondria causes mitochondrial swelling and ROS production, as well as disruption of mitochondrial structure (as found in this study) by irreversibly opening the mitochondrial permeability transition pore (mPTP) [48], which in turn lead to mitochondrial dysfunction [49].

### 3.5. The Arrhythmias in DMD Cardiomyocytes

Among the variety of DMD-related deficits presented here, the arrhythmias in DMD adult and 13y CMs were likely triggered by intracellular Ca^2+^-overload, extensively reported to occur in DMD CMs. Similarly, Kamdar and co-workers [50] reported that, at baseline and following adrenergic stimulation, DMD iPSC-CM presented arrhythmic calcium traces. In this regard, several studies have reported on aberrations in intracellular Ca^2+^ handling in DMD CMs [51,52,53,54,55]. Briefly, we recently found that, compared to healthy cells, DMD adult male and female iPSC-CMs exhibited a blunted positive inotropic response to β-adrenergic stimulation by isoproterenol [54]. In support of the hypothesis that this was due to depleted SR Ca^2+^ stores, which may be caused by an SR RyR leak [55], resulting in cytosolic Ca^2+^-overload (which underlies DADs), we found that, compared to healthy cells, DMD CMs exhibited reduced caffeine-induced Ca^2+^ SR release and an attenuated negative inotropic response to ryanodine and cyclopiazonic acid. Further, cytosolic Ca^2+^ overload, a primary mechanism underlying DAD generation and conduction abnormalities, can be caused by bioenergetic/metabolic deficits, along with reduced ATP depletion as found in DMD CMs (Figure 1 and Figure 4).

### 3.6. Do Fibroblasts “Remember” the Age of the DMD Patient?

Our findings from the 7y CMs deserve special attention; while these CMs present some adult DMD cardiomyocyte abnormalities—increased mitochondrial density and slow spontaneous firing rate—they do not present the other adult CMs abnormalities, such as prominent arrhythmias, decreased PCr, aberrant mitochondrial morphology and impaired oxidative phosphorylation. Although the CMs were derived via iPSC from skin fibroblasts, they “epitomize” the clinical state of the patient from which they were obtained. Indeed, the 7y male was by far less sick than the 13y male, the 32 year old male and the 50 year old female, the latter two suffering from prominent DCM. Hence, a fundamental unresolved question is at what age the presence of the *dystrophin* mutation is translated into/manifested as the diverse pathologies, such as bioenergetic deficits and cardiac arrhythmias. The clinical status of the DMD 7y male (and the majority of young DMD patients), as well as the healthy-like behavior of the 7y iPSC-CMs, corresponded to the mild cardiac disease in young (1–2-month-old) *mdx* mice resulting from cardiac adaption mechanisms, largely residing in the mitochondria. Indeed, several reports have shown that the cardiac mitochondria from young *mdx* mice do not differ from wild-type mice with respect to respiration and H_2_O_2_ generation (for example, [56,57]). Specifically, Dubinin et al., reported that, as compared to wild-type animals, heart mitochondria of *mdx* mice have been found to be more efficient both with respect to Ca^2+^ uniport and Na^+^-dependent Ca^2+^ efflux. The data obtained indicate that the increased rate of Ca^2+^ uptake by heart mitochondria in young *mdx* mice may be due to an increase in the ratio of MCU and MCU subunits [56].

In conclusion, based on our novel findings, we propose that the *dystrophin* mutations responsible for a dysfunctional dystrophin protein affect in turn mitochondrial function in the dystrophic myofibers, causing impaired oxidative phosphorylation and low PCr levels over time. Our study strongly supports the notion that iPSC-CMs are a trustworthy model for DMD disease, both according to electrophysiological and metabolic criteria and from the finding that DMD is manifested by mitochondrial dysfunction.

## 4. Materials and Methods

### 4.1. The Experimental Groups

Dermal biopsies were obtained from four DMD patients. (1) A 32-year-old male DMD patient carrying a substitution of cytosine to thymine (c.5899C > T) constituting a premature stop codon (AM DMD). (2) A 50-year-old female DMD patient carrying a deletion of exons 8–12 (ex.8_12del). Patients 1 and 2 were included in our recent publication [29]; skin biopsies from these patients were obtained after the patients signed an informed consent form (approval #7603-09-SMC by the Helsinki Committee for Experiments on Human Subjects at Sheba Medical Center, Ramat Gan, Israel). (3) A 7-year-old (7y) male DMD patient carrying a nonsense mutation (c.8038C > T) that led to premature termination of translation and a truncated dystrophin protein. (4) A 13-year-old (13y) male DMD patient lacking exons 45–50 of the dystrophin gene (IITi001-A) [58]. The donor signed a consent form in accordance with the approval IRB HUM00030934 from the University of Michigan Committee. As controls, we used: (1) an FSE-5m clone generated from healthy neonatal foreskin fibroblasts, as previously described [59]; (2) a 24.5 clone generated from a healthy 42-year-old female, as previously characterized and described [60]. This investigation conformed to the principles outlined in the Declaration of Helsinki.

### 4.2. Experimental Protocols

See the Supplementary Materials for details on the patients’ clinical histories, iPSC generation, karyotype analysis (the 7y, Supplementary Figure S1), genotyping (the 7y, Figure S1), teratoma formation (7y, Supplementary Figure S1), differentiation into cardiomyocytes, action potential recordings, the Seahorse Flux Analyzer, fluorescence measurements of mitochondrial area and activity, metabolomics and transmission electron microscopy (TEM).

### 4.3. Statistical Analysis

A detailed description of the statistical analysis applied for each type of experiment is provided in the figure captions and in the Methods section in the Supplementary Materials. Results are presented as means ± the standard error of the mean (SEM). A value of *p* < 0.05 was considered statistically significant, where (*) represents *p* < 0.05, (**) represents *p* < 0.01, (***) represents *p* < 0.001 and (****) represents *p* < 0.0001.

## Data Availability

Data were uploaded to the Metabolights depository and will be publicly available in the Metabolights team revision at https://www.ebi.ac.uk/metabolights/editor/www.ebi.ac.uk/metabolights/MTBLS5343 (accessed on 11 July 2022).

## Supplementary Materials

The following supporting information can be downloaded at: https://www.mdpi.com/article/10.3390/ijms23179808/s1. Refer to Refs. [29,33,58,59,60,61,62,63,64,65,66,67,68].

## References

[1] Mavrogeni S., Markousis-Mavrogenis G., Papavasiliou A., Kolovou G. (2015). Cardiac involvement in Duchenne and Becker muscular dystrophy. World J. Cardiol..

[2] Bushby K., Finkel R., Birnkrant D.J., Case L.E., Clemens P.R., Cripe L., Kaul A., Kinnett K., McDonald C., Pandya S. (2010). Diagnosis and management of Duchenne muscular dystrophy, part 2: Implementation of multidisciplinary care. Lancet Neurol..

[3] Eagle M., Baudouin S.V., Chandler C., Giddings D.R., Bollock R., Bushby K. (2002). Survival in Duchenne muscular dystrophy: Improvements in life expectancy since 1967 and the impact of home nuctural ventilation. Neuromuscul. Disord..

[4] McNally E.M., Kaltman J.R., Benson D.W., Canter C.E., Cripe L.H., Duan D., Finder J.D., Groh W.J., Hoffman E.P., Judge D.P. (2015). Contemporary cardiac issues in Duchenne muscular dystrophy. Circulation.

[5] Allen D.G., Whitehead N.P., Froehner S.C. (2016). Absence of Dystrophin Disrupts Skeletal Muscle Signaling: Roles of Ca ^2+^, Reactive Oxygen Species, and Nitric Oxide in the Development of Muscular Dystrophy. Physiol. Rev..

[6] D’Amario D., Amodeo A., Adorisio R., Tiziano F.D., Leone A.M., Perri G., Bruno P., Massetti M., Ferlini A., Pane M. (2017). A current approach to heart failure in Duchenne muscular dystrophy. Heart.

[7] Rajdev A., Groh W.J. (2015). Arrhythmias in the muscular dystrophies. Card. Electrophysiol. Clin..

[8] Finsterer J., Stöllberger C. (2000). Cardiac involvement in primary myopathies. Cardiology.

[9] Finsterer J., Stollberger C. (2003). The heart in human dystrophinopathies. Cardiology.

[10] Kamdar F., Garry D.J. (2016). Dystrophin-Deficient Cardiomyopathy. J. Am. Coll. Cardiol..

[11] Ware S.M. (2017). Genetics of paediatric cardiomyopathies. Curr. Opin. Pediatr..

[12] Omairi S., Hau K.L., Collins-Hooper H., Scott C., Vaiyapuri S., Torelli S., Montanaro F., Matsakas A., Patel K. (2019). Regulation of the dystrophin-associated glycoprotein complex composition by the metabolic properties of muscle fibres. Sci. Rep..

[13] Timpani C.A., Hayes A., Rybalka E. (2015). Revisiting the dystrophin-ATP connection: How half a century of research still implicates mitochondrial dysfunction in Duchenne muscular dystrophy aetiology. Med. Hypotheses.

[14] Gumerson J.D., Michele D.E. (2011). The dystrophin-glycoprotein complex in the prevention of muscle damage. J. Biomed. Biotechnol..

[15] Hughes M.C., Ramos S.V., Turnbull P.C., Rebalka I.A., Cao A., Monaco C.M.F., Varah N.E., Edgett B.A., Huber J.S., Tadi P. (2019). Early myopathy in Duchenne muscular dystrophy is associated with elevated mitochondrial H_2_O_2_ emission during impaired oxidative phosphorylation. J. Cachexia Sarcopenia Muscle.

[16] Esposito G., Carsana A. (2019). Clinical Medicine Metabolic Alterations in Cardiomyocytes of Patients with Duchenne and Becker Muscular Dystrophies. J. Clin. Med..

[17] Groh W.J. (2012). Arrhythmias in the muscular dystrophies. Heart Rhythm.

[18] Yilmaz A., Sechtem U. (2012). Cardiac involvement in muscular dystrophy: Advances in diagnosis and therapy. Heart.

[19] Rybalka E., Timpani C.A., Cooke M.B., Williams A.D., Hayes A. (2014). Defects in mitochondrial ATP synthesis in dystrophin-deficient Mdx skeletal muscles may be caused by complex I insufficiency. PLoS ONE.

[20] Gambardella J., Trimarco B., Iaccarino G., Santulli G. (2018). New Insights in Cardiac Calcium Handling and Excitation-Contraction Coupling. Adv. Exp. Med. Biol..

[21] Gambardella J., Sorriento D., Ciccarelli M., Del Giudice C., Fiordelisi A., Napolitano L., Trimarco B., Iaccarino G., Santulli G. (2017). Functional Role of Mitochondria in Arrhythmogenesis. Adv. Exp. Med. Biol..

[22] Lindsay A., Chamberlain C.M., Witthuhn B.A., Lowe D.A.l., Ervast J.M. (2019). Dystrophinopathy-associated dysfunction of Krebs cycle metabolism. Hum. Mol. Genet..

[23] Ramos S.V., Hughes M.C., Delfinis L.J., Bellissimo C.A., Perry C.G.R. (2020). Mitochondrial bioenergetic dysfunction in the D2.mdx model of Duchenne muscular dystrophy is associated with microtubule disorganization in skeletal muscle. PLoS ONE.

[24] Chang A.C.Y., Ong S.G., LaGory E.L., Kraft P.E., Giaccia A.J., Wu J.C., Blau H.M. (2016). Telomere shortening and metabolic compromise underlie dystrophic cardiomyopathy. Proc. Natl. Acad. Sci. USA.

[25] Stapleton D.I., Lau X., Flores M., Trieu J., Gehrig S.M., Chee A., Naim T., Lynch G.S., Koopman R. (2014). Dysfunctional muscle and liver glycogen metabolism in mdx dystrophic mice. PLoS ONE.

[26] Strakova J., Kamdar F., Kulhanek D., Razzoli M., Garry D.J., Ervasti J.M., Bartolomucci A., Townsend D. (2018). Integrative effects of dystrophin loss on metabolic function of the mdx mouse. Sci. Rep..

[27] McConell G.K., Rattigan S., Lee-Young R.S., Wadley G.D., Merry T.L. (2012). Skeletal muscle nitric oxide signaling and exercise: A focus on glucose metabolism. AJP Endocrinol. Metab..

[28] Cole M.A., Rafael J.A., Taylor D.J., Lodi R., Davies K.E., Styles P.A. (2002). Quantitative study of bioenergetics in skeletal muscle lacking utrophin and dystrophin. Neuromuscul. Disord..

[29] Eisen B., Ben Jehuda R., Cuttitta A.J., Mekies L.N., Shemer Y., Baskin P., Reiter I., Willi L., Freimark D., Gherghiceanu M. (2019). Electrophysiological abnormalities in induced pluripotent stem cell-derived cardiomyocytes generated from Duchenne muscular dystrophy patients. J. Cell. Mol. Med..

[30] Sala L., Bellin M., Mummery C.L. (2017). Integrating cardiomyocytes from human pluripotent stem cells in safety pharmacology: Has the time come?. Br. J. Pharmacol..

[31] Neal R.C., Ferdinand K.C., Yčas J., Miller E. (2009). Relationship of ethnic origin, gender, and age to blood creatine kinase levels. Am. J. Med..

[32] Ben-Ari M., Naor S., Zeevi-Levin N., Schick R., Ben Jehuda R., Reiter I., Raveh A., Grijnevitch I., Barak O., Rosen M.R. (2016). Developmental changes in electrophysiological characteristics of human-induced pluripotent stem cell-derived cardiomyocytes. Heart Rhythm.

[33] Ben Jehuda R., Eisen B., Shemer Y., Mekies L.N., Szantai A., Reiter I., Cui H., Guan K., Haron-Khun S., Freimark D. (2017). CRISPR correction of the PRKAG2 gene mutation in the patient’s iPSC-derived cardiomyocytes eliminates the electrophysiological and structural abnormalities. Heart Rhythm.

[34] Piquereau J., Caffin F., Novotova M., Lemaire C., Veksler V., Garnier A., Ventura-Clapier R., Joubert F. (2013). Mitochondrial dynamics in the adult cardiomyocytes: Which roles for a highly specialized cell?. Front. Physiol..

[35] Burelle Y., Khairallah M., Ascah A., Allen B.G., Deschepper C.F., Petrof B.J., Des Rosiers C. (2010). Alterations in mitochondrial function as a harbinger of cardiomyopathy: Lessons from the dystrophic heart. J. Mol. Cell. Cardiol..

[36] Kushmerick M.J. (1995). Skeletal muscle: A paradigm for testing principles of bioenergetics. J. Bioenerg. Biomembr..

[37] Kushmerick M.J. (1985). Patterns in mammalian muscle energetics. J. Exp. Biol..

[38] Kushmerick M.J. (1987). Energetics studies of muscles of different types. Basic Res. Cardiol..

[39] Pulido S.M., Passaquin A.C., Leijendekker W.J., Challet C., Wallimann T., Rüegg U.T. (1998). Creatine supplementation improves intracellular calcium handling and survival in mdx skeletal muscle cells. FEBS Lett..

[40] Zhang W., ten Hove M., Schneider J.E., Stuckey D.J., Sebag-Montefiore L., Bia B.L., Radda G.K., Davies K.E., Neubauer S., Clarke K. (2008). Abnormal cardiac morphology, function and energy metabolism in the dystrophic mdx mouse: An MRI and MRS study. J. Mol. Cell. Cardiol..

[41] Moore T.M., Lin A.J., Strumwasser A.R., Strumwasser A.R., Cory K., Whitney K., Ho T., Ho T., Lee J.L., Rucker D.H. (2020). Mitochondrial Dysfunction Is an Early Consequence of Partial or Complete Dystrophin Loss in mdx Mice. Front. Physiol..

[42] Kang C., Badr M.A., Kyrychenko V., Eskelinen E.L., Shirokova N. (2018). Deficit in PINK1/PARKIN-mediated mitochondrial autophagy at late stages of dystrophic cardiomyopathy. Cardiovasc. Res..

[43] Onopiuk M., Brutkowski W., Wierzbicka K., Wojciechowska S., Szczepanowska J., Fronk J., Lochmüller H., Górecki D.C., Zabłocki K. (2009). Mutation in dystrophin-encoding gene affects energy metabolism in mouse myoblasts. Biochem. Biophys. Res. Commun..

[44] Giacomotto J., Brouilly N., Walter L., Mariol M.C., Berger J., Ségalat L., Becker T.S., Currie P.D., Gieseler K. (2013). Chemical genetics unveils a key role of mitochondrial dynamics, cytochrome c release and IP3R activity in muscular dystrophy. Hum. Mol. Genet..

[45] Dubinin M.V., Starinets V.S., Talanov E.Y., Mikheeva I.B., Belosludtseva N.V., Serov D.A., Tenkov K.S., Belosludtseva E.V., Belosludtsev K.N. (2021). Effect of the non-immunosuppressive mpt pore inhibitor alisporivir on the functioning of heart mitochondria in dystrophin-deficient mdx mice. Biomedicines.

[46] Mareedu S., Million E.D., Duan D., Babu G.J. (2021). Abnormal calcium handling in Duchenne muscular dystrophy: Mechanisms and potential therapies. Front. Physiol..

[47] Zabłocka B., Górecki D.C., Zabłocki K. (2021). Disrupted calcium homeostasis in Duchenne muscular dystrophy: A common mechanism behind diverse consequences. Int. J. Mol. Sci..

[48] Reid A.L., Alexander M.S. (2021). The interplay of mitophagy and inflammation in Duchenne muscular dystrophy. Life.

[49] Bellissimo C.A., Garibotti M.C., Perry C.G.R. (2022). Mitochondrial stress responses in Duchenne muscular dystrophy: Metabolic dysfunction or adaptive reprogramming?. Am. J. Physiol. Cell Physiol..

[50] Kamdar F., Das S., Gong W., Kamdar A., Meyers T.A., Shah P., Ervasti J.M., Townsend D., Kamp T.J., Wu J.C. (2020). Stem Cell–derived cardiomyocytes and beta-adrenergic receptor blockade in Duchenne Muscular Dystrophy cardiomyopathy. J. Am. Coll. Cardiol..

[51] Dubinin M.V., Talanov E.Y., Tenkov K.S., Starinets V.S., Mikheeva I.B., Sharapov M.G., Belosludtsev K.N. (2020). Duchenne muscular dystrophy is associated with the inhibition of calcium uniport in mitochondria and an increased sensitivity of the organelles to the calcium-induced permeability transition. Biochim. Biophys. Acta Mol. Basis Dis..

[52] Law M.L., Cohen H., Martin A.A., Angulski A.B.B., Metzger J.M. (2020). Dysregulation of calcium handling in duchenne muscular dystrophy-associated dilated cardiomyopathy: Mechanisms and experimental therapeutic strategies. J. Clin. Med..

[53] Farini A., Sitzia C., Cassinelli L., Colleoni F., Parolini D., Giovanella U., Maciotta S., Colombo A., Meregalli M., Torrente Y. (2016). Inositol 1,4,5-trisphosphate (IP3)-dependent Ca^2+^ signaling mediates delayed myogenesis in Duchenne muscular dystrophy fetal muscle. Development.

[54] Mekies L.N., Regev D., Eisen B., Fernandez-Gracia J., Baskin P., Ben Jehuda R., Shulman R., Reiter I., Palty R., Arad M. (2021). Depressed β-adrenergic inotropic responsiveness and intracellular calcium handling abnormalities in Duchenne Muscular Dystrophy patients’ induced pluripotent stem derived cardiomyocytes. J. Cell Mol. Med..

[55] Fauconnier J., Thireau J., Reiken S., Cassan C., Richard S., Matecki S., Marks A.R., Lacampagne A. (2010). Leaky RyR2 trigger ventricular arrhythmias in Duchenne muscular dystrophy. Proc. Natl. Acad. Sci. USA.

[56] Dubinin M.V., Talanov E.Y., Tenkov K.S., Talanov E.Y., Starinets V.S., Tenkov K.S., Zakharova N.M., Belosludtseva N.V. (2020). Transport of calcium and calcium-dependent permeability transition in heart mitochondria in the early stages of Duchenne muscular dystrophy. Biochim. Biophys. Acta Bioenerg..

[57] Ascah A., Khairallah M., Daussin F., Bourcier-Lucas C., Godin R., Allen B.G., Petrof B.J., Des Rosiers C., Burelle Y. (2011). Stress-induced opening of the permeability transition pore in the dystrophin-deficient heart is attenuated by acute treatment with sildenafil. Am. J. Physiol. Hearth Circ. Physiol..

[58] Eisen B., Ben Jehuda R., Cuttitta A.J., Mekies L.N., Reiter I., Ramchandren S., Arad M., Michele D.E., Binah O. (2018). Generation of Duchenne muscular dystrophy patient-specific induced pluripotent stem cell line lacking exons 45–50 of the dystrophin gene (IITi001-A). Stem Cell Res..

[59] Yehezkel S., Rebibo-Sabbah A., Segev Y., Tzukerman M., Shaked R., Huber I., Gepstein L., Skorecki K., Selig S. (2011). Reprogramming of telomeric regions during the generation of human induced pluripotent stem cells and subsequent differentiation into fibroblast-like derivatives. Epigenetics.

[60] Novak A., Barad L., Lorber A., Gherghiceanu M., Reiter I., Eisen B., Eldor L., Itskovitz-Eldor J., Eldar M., Arad M. (2015). Functional abnormalities in iPSC-derived cardiomyocytes generated from CPVT1 and CPVT2 patients carrying ryanodine or calsequestrin mutations. J. Cell. Mol. Med..

[61] Jimenez-Vazquez E.N., Arad M., Macías Á., Vera-Pedrosa L., Cruz-Uréndez F.M., Cuttitta A.J., Monteiro Da Rocha A., Herron T.J., Ponce-Balbuena D., Guerrero-Serna G. (2022). SNTA1 Gene rescues ion channel function in cardiomyocytes derived from induced Pluripotent Stem Cells reprogrammed from Muscular Dystrophy patients with arrhythmias. eLife.

[62] Streckfuss-Bömeke K., Wolf F., Azizian A., Stauske M., Tiburcy M., Wagner S., Hübscher D., Dressel R., Chen S., Jende J. (2013). Comparative study of human-induced pluripotent stem cells derived from bone marrow cells, hair keratinocytes, and skin fibroblasts. Eur. Heart J..

[63] Novak A., Barad L., Zeevi-Levin N., Shick R., Shtrichman R., Lorber A., Itskovitz-Eldor J., Binah O. (2012). Cardiomyocytes generated from CPVT D307H patients are arrhythmogenic in response to betha-adrenergic stimulation. J. Cell Mol. Med..

[64] MacKay G.M., Zheng L., Broek N.J.F., Van Den Gottlieb E. (2015). Analysis of cell metabolism using LC-MS and isotope tracers. Methods Enzymol..

[65] Anders S., Huber W. (2010). Differential expression analysis for sequence count data. Genome Biol..

[66] Pietzke M., Vazquez A. (2020). Metabolite AutoPlotter—An application to process and visualise metabolite data in the web browser. Cancer Metab..

[67] Ben-Ari M., Schick R., Barad L., Novak A., Ben-Ari E., Lorber A., Itskovitz-Eldor J., Rosen M.R., Weissman A., Binah O. (2014). From beat rate variability in induced pluripotent stem cell-derived pacemaker cells to heart rate variability in human subjects. Heart Rhythm..

[68] Mandel Y., Weissman A., Schick R., Barad L., Novak A., Meiry G., Goldberg S., Lorber A., Rosen M.R., Itskovitz-Eldor J. (2012). Human embryonic and induced pluripotent stem cell-derived cardiomyocytes exhibit beat rate variability and power-law behavior. Circulation.

