# Supplementary material for: Bioenergetic and Metabolic Impairments in Induced Pluripotent Stem Cell-Derived Cardiomyocytes Generated from Duchenne Muscular Dystrophy Patients
Source: Int J Mol Sci. 2022 Aug 29;23(17):9808. doi: 10.3390/ijms23179808 (PMC9456153; doi:10.3390/ijms23179808)
Supplement: Supplementary file 1 [file ijms-23-09808-s001.zip › ijms-1835810-supplementary.pdf]

International Journal of *Molecular Sciences*

MDPI

*Article*

# Bioenergetic and Metabolic Impairments in Induced Pluripotent Stem Cell-Derived Cardiomyocytes Generated from Duchenne Muscular Dystrophy Patients

**Lubna Willi [1,†], Ifat Abramovich [2,†], Jonatan Fernandez-Garcia [2], Bella Agranovich [2], Margarita Shulman [1], Helena Milman [1], Polina Baskin [1], Binyamin Eisen [1], Daniel E. Michele [3], Michael Arad [4,5], Ofer Binah [1,*] and Eyal Gottlieb [2,*]**

1 Department of Physiology, Biophysics and Systems Biology, Rappaport Faculty of Medicine and Research Institute, Technion, Haifa 31096, Israel
2 Department of Cell Biology and Cancer Science, Rappaport Faculty of Medicine and Research Institute, Technion, Haifa 31096, Israel
3 Department of Molecular and Integrative Physiology, University of Michigan, Ann Arbor, MI 48109, USA
4 Leviev Heart Center, Sheba Medical Center, Ramat Gan 52621, Israel
5 Sackler Faculty of Medicine, Tel Aviv University, Tel Aviv 69978, Israel
* Correspondence: binah@technion.ac.il (O.B.); e.gottlieb@technion.ac.il (E.G.)
† These authors contributed equally to this work.

## Supplement Methods

### Clinical history of the DMD patients

The study includes 4 DMD patients: (1) a DMD 7-year-old (7y) male presenting with early stages of muscle weakness, without any DCM diagnosis; (2) a 32-year-old adult male who suffered from muscular dystrophy from early childhood and was diagnosed with DCM at the age of 17[1]; (3) a 50-year-old adult female manifesting carrier patient who presented at age 42 with proximal muscle weakness and creatine kinase elevation[1], and a year later was diagnosed with DCM. The patient expired at age 51 because of end-stage heart failure associated with renal insufficiency. Her son who was diagnosed with DMD expired at age 16. (4) A 13-year-old (13y) male – which at the time of the biopsy was wheel-chair bound but without recorded evidence of heart disease[2]. Detailed clinical information regarding patients 2 and 3 is pesented in Jimenez-Vazquez et al, 2022[3].

**Generation of induced Pluripotent Stem Cells (iPSCs) from the DMD patients and healthy male and female volunteers**

iPSCs from the DMD *7y* male were generated from the patient's dermal fibroblasts using Sendai virus CytoTune-iPS 2.0 Reprogramming Kit, #A16517 (Thermo Fisher, Waltham, MA, USA)[1,4]. As routinely done with new patients, skin fibroblasts were reprogramed into iPSCs, karyotyped, genotyped and tested for the ability to generate teratoma (Figure S1). As control, we used the following healthy clones: clone FSE-5m generated from healthy neonatal foreskin fibroblasts as previously described[5]; clone 24.5 generated from a healthy 42-year-old female as previously characterized and described[6] using the STEMCCA system, containing all four reprogramming factors OCT4, SOX2, KLF4 and c-MYC in a single 'stem cell cassette' (pHAGE2-EF1aFull-hOct4-F2AhKlf4-IRES-hSox2-P2A-hcMyc-W-loxP)[7].

**Karyotype analysis**

Karyotype analysis was conducted according to standard procedures as previously described[8].

**Genotyping**

To confirm the mutation in young male DMD was preserved in the iPSC and iPSC-CM clones, genomic DNA was isolated from the cells using QIAGEN DNeasy Blood & Tissue Kit (QIAGEN, Hilden, Germany) and PCR was performed to exon 55 of the dystrophin gene using the following primer sequences: Forward AGTTCCTCCATCTTTCTCTTTTTATG, Reverse

TGTTTTGTCCCTGGCTTGTCA, resulting in a product of 372 base pairs in length. Next, the PCR product was purified using QIAquick PCR Purification Kit (QIAGEN, Hilden, Germany), followed by Sanger sequencing from both ends of the PCR product containing the mutation area within the dystrophin gene. Subsequently, we confirmed the nonsense mutation (substitution of cytosine to thymine) in exon 55 leading to the replacement of arginine with a premature termination codon.

**Teratoma Formation**

To determine the iPSCs differentiation capacity *in vivo*, iPSCs colonies from two Matrigel coated 6-well plates in mTeSR1 medium (Stemcell Technologies, Vancouver, Canada) were washed 3 times in PBS and injected into the thigh muscle of severe combined immunodeficient (SCID) mice. Teratomas were observed 8-12 weeks after injection, and images were obtained from formalin-fixed (4%) and paraffin-embedded teratoma sections stained with hematoxylin and eosin (H&E).

**Differentiation into cardiomyocytes**

iPSC-CMs were generated according to the directed differentiation by modulating Wnt/β-catenin signaling as previously described[5]. iPSCs were cultured on Matrigel (GFR, BD Biosciences, Franklin Lakes, NJ, USA) coated 6-well plates in mTeSR1 medium (Stemcell Technologies, Vancouver, Canada) for 5-6 days. To initiate differentiation, cells were incubated with 1 ml/well Versene solution (Invitrogen, Life Technologies, Woburn, MA, USA) at 37°C for 7 minutes and seeded on Matrigel coated plate at $8.5 \times 10^6$/12 well plate density in mTeSR1 medium supplemented with 5 µmol/l ROCK inhibitor (Cayman Chemical, Ann Arbor, MI, USA). The medium was replaced daily, and after 2 days when the monolayer of cells reached 100% confluence, the

medium was changed to RPMI supplemented with B27 minus insulin (Invitrogen, Life Technologies, Woburn, MA, USA) containing 8 or 10 µmol/l CHIR99021 and this day was labeled as day 1 of differentiation. On the next day (day 2 of differentiation), the medium was changed to RPMI supplemented with B27 minus insulin. On day 4, the medium was changed to RPMI supplemented with B27 minus insulin, containing 5 or 10 µmol/l of IWP-4. On day 6, the medium was changed to RPMI supplemented with B27 minus insulin. Finally, from day 8 onwards, the medium was changed to RPMI supplemented with B27 complete supplement (Invitrogen, Life Technologies, Woburn, MA, USA).

**Metabolomics experiments and carbon-13 stable isotope tracing experiments**

All metabolomic studies were performed as previously described with minor differences[9].

Sample preparation for metabolomic analysis

iPSC-CMs ($1x10^6$) were seeded on a solidified Matrigel (6-well plate, 200 µl/9.6 $cm^2$) in RPMI 1640 medium. For metabolomics experiments the media was changed to Advanced modified DMEM F12 (without glucose, glutamine and pyruvate to enable stable isotope tracing experiments). The media was supplemented with glucose (5 mM), glutamine (0.65 mM), pyruvate (0.1 mM), carnitine (0.046 mM), creatine (0.037 mM) and taurine (0.13 mM) for a 24 hrs adaptation. After the adaptation period, the cells were incubated with media containing the same supplement

concentration with 5 mM $^{13}C_6$-labeled glucose. After 24-h incubation at 37°C, metabolites were extracted as described below.

Extracellular metabolite extraction: Fifty µl of supernatant were mixed with 950 µl of cold (-20°C) methanol/acetonitrile/water (5:3:2 v/v/v) extraction buffer, mixed for 10 min at 4°C, and spun 10 min at 16,100 × g at 4°C. Eight hundred µl of the cleared supernatant were transferred to glass HPLC vials and stored at −80°C until LC-MS analysis.

Intracellular metabolite extraction: After removal of the media, cells were quickly washed with cold PBS. A pre-chilled recovery solution (Corning Recovery Solution, Cat #FAL354253) was added and each sample was gently pipetted up and down using wide bore tips to carefully break up the Matrigel matrix. After 20 minutes incubation at 4°C for 20 minutes, the cells were gently centrifuged at 2000 rpm for 5 minutes. The dissolved Matrigel layer was discarded, the cells were washed with PBS and metabolites extracted upon incubation with 500 µl of cold extraction buffer for 10 min at 4°C. The sup with metabolites was collected and centrifuged for 10 min at 16,100 × g at 4°C. Two hundrad µl of the cleared supernatant were transferred to HPLC glass vial equipped with insert and stored at −80°C until LC-MS analysis.

Metabolomics LC-MS Analysis: Matabolomics analysis was conducted as described privously[9] . Briefly: Thermo Ultimate 3000 high-performance liquid chromatography (HPLC) system coupled to Q- Exactive Orbitrap Mass Spectrometer (Thermo Fisher Scientific) was used with a resolution of

35,000 at 200 mass/charge ratio (m/z), electrospray ionization, and polarity switching mode to enable both positive and negative ions across a mass range of 67-to-1000 m/z. HPLC setup consisted ZIC-pHILIC column (SeQuant; 150 mm x 2.1 mm, 5 μm; Merck), with a ZIC-pHILIC guard column (SeQuant; 20 mm x 2.1 mm). Five μl of biological extracts were injected and the compounds were separated with a mobile phase gradient of 15 min, starting at 20% aqueous (20 mM ammonium carbonate adjusted to pH = 2, with 0.1% of 25% ammonium hydroxide) together with 80% organic (acetonitrile) and terminated with 20% acetonitrile. Flow rate and column temperature were maintained at 0.2 ml/min and 45°C respectively, for a total run time of 27 min. All metabolites were detected using mass accuracy below 5 ppm. Thermo Xcalibur was used for data acquisition.

_Metabolomics Data Analysis_: For untargeted analysis, raw data were analysed using Compound Discoverer software (Thermo Scientific v3.2). Retention times were aligned across all sample data files (maximum shift 2 min, mass tolerance 5 ppm). Unknown compound detection (minimum peak intensity $10^5$) and grouping of compound adducts was carried out across all samples (mass tolerance 5 ppm, RT tolerance 0.2 min). Missing values were filled using software's Fill Gap feature (mass tolerance 5 ppm, S/N tolerance 1.5). TraceFinder 5.1 was used for targeted data analysis. Peak areas (= measured intensity) of metabolites were determined by using the exact mass of the singly charged ions. The retention time of identified metabolites was predetermined on the pHILIC column by analyzing an in-house mass spectrometry metabolite library that was built by running commercially available standards. $^{13}C$ labeling patterns were determined by measuring peak areas for the accurate mass of each isotopologues. Each metabolite peak area value analyzed in the

samples, was normalized to median of ratios[10]. Data analysis processing and visualization was done using Metabolite AutoPlotter[11] .

## Electrophysiological experiments

Action potentials (APs) were recorded from the iPSC-CMs in whole-cell configuration. Small clusters were plated on Matrigel-coated (GFR, BD Biosciences, Franklin Lakes, NJ, USA) glass coverslips (13 mm diameter) in 24-well plates. The coverslips were incubated at 37°C, and a recovery period of at least two days was allowed before performing electrophysiological experiments[12,13]. In all experiments, the coverslips were perfused at 37°C with an external solution containing (in mM): 140 NaCl, 5.4 KCl, 1.8 $CaCl_2$, 1 $MgCl_2$, 10 glucose and 10 HEPES titrated to pH 7.4 with NaOH (310 mOsm). The patch pipette solution contained (mM): 120 KCl, 1 $MgCl_2$, 3 Mg-ATP, 10 HEPES, and 10 EGTA titrated to pH 7.2 with KOH and adjusted at 290 mOsm with saccharose (all materials were purchased from Sigma-Aldrich). Axopatch 200B, Digidata 1322 and pClamp10 (Molecular Devices, Sunnyvale, CA, USA) were used for data amplification, acquisition and analysis. Signals were digitized at 10 kHz and filtered at 2 kHz. Microelectrodes with resistances of 4-7 MΩ were pulled from borosilicate glass capillaries (Harvard Apparatus, Holliston, MA, USA). Dedicated MATLAB software was used to analyse the recordings for the detection of all peaks of the recorded signal for action potential parameters and to calculate IBIs[13] .

## Transmission electron microscopy (TEM) of iPSC-CMs

Samples were fixed with 2.5% glutaraldehyde and 3% parafalmaldehyde in 0.1 M Cacodylate buffer. Fixed iPSC-CM clusters were embedded in 3.4% agar, post-fixed for 50 min in buffered 1%

$OsO_4$ + 5mM $CaCl_2$ + 0.5% Potassium dichromate ($K_2Cr_2O_7$) + 0.5% potassium hexacyonaferrate ($K_4[Fe(CN)_6]$). Subsequently, the samples were dehydrated in graded ethanol series and embedded in epoxy resin (EMBED 812/DER 736 KIT, EMS). Seventy nm sections were cut with diatom diamond knife using a Leica UC7 ultramicrotome and transferred to copper grids. The grids were coated with a thin layer of carbon to increase conductivity, and examined using Zeiss Ultra-Plus FEG-SEM equipped with STEM detector at accelerating voltage of 30 kV.

**Mitochondrial potential measurements**

iPSC-CMs ($2x10^5$) were obtained from a healthy male donor and 3 DMD patients: DMD adult male, DMD adult female and 7y male. CMs were each seeded onto a 35 mm glass-bottom dish (Fluorodish, World Precision Instruments, Sarasota, FL, USA) and grown for one week in RPMI culture medium (Invitrogen, Life Technologies, Woburn, MA, USA) with B27 insulin supplement (Invitrogen, Life Technologies, Woburn, MA, USA). On the day of the experiment the medium was replaced with serum-free EB medium supplemented with 50 nmol/L Mitotracker Green (MTG) (Invitrogen, Thermo Fisher Scientific) and 10 nmol/L Tetramethyl Rhodamine Ethyl Ester (TMRE) (Thermo Fisher Scientific) for 30 minutes at $37^oC$. The final concentrations of the drugs were: oligomycin 2 μmol/l, FCCP 10 μmol/L. Beating CMs were imaged using the Zeiss LSM 700 confocal microscope built on a Axio-Observer inverted microscope equipped with a 405, 488 and 555 solid state lasers using a 63x1.4NA Plan apochromat objective. A whole cell fluorescence image z-stack (z-step was set at 0.4 μm) was acquired. Mitochondrial three-dimensional (3D) reconstruction was

achieved using the segmentation algorithm of Imaris 9.2 (Bitplane, Zurich, Switzerland). The ratio of TMRE volume over MTG was calculated to represent the mitochondrial potential.

**Oxygen consumption rate (OCR) and extracellular acidification rate (ECAR)**

Experiments were performed in a 96-well format using an Agilent Seahorse XFe96 Extracellular Flux Analyzer. DMD CMs ($3\times10^4$) and $3\times10^4$ healthy control CMs were plated on Matrigel pre-coated XF96 plates and incubated at 37°C, 5% $CO_2$ for 24 hr. The medium was then replaced with 180 µl of unbuffered assay media (Sigma D5030) supplemented with 10 mM glucose, 1 mM pyruvate and 2 mM glutamine (pH 7.4) for Mitochondrial Stress Test, or 2 mM glutamine only for glycolysis stress test. CMs were then placed at 37°C in a $CO_2$-free incubator for 45 minutes. During the experiment, 1 µM oligomycin A (Sigma), 1.0 µM FCCP (Sigma) and 50 µM rotenone and antimycon A mixture (Sigma) were injected sequentially. For Glycolysis Stress test, the assay medium was supplemented with 2 mM glutamine. The CMs were deprived of glucose for 1 hr. During the experiment, 10 mM glucose (Sigma), 1 µM oligomycin A (sigma) and 50 mM of 2-Deoxyglucose (Sigma) were injected sequentially. OCR and ECAR were normalized to the protein content in each well calculated at the end of the experiments by using Modified Lowry protein quantitation assay. Data were analyzed using Wave software version 2.6.

**Statistical analysis**

Comparisons between the experimental groups in the different experiments were subjected to normality test before deciding on the proper statistical analysis. The applied statistical analysis

was performed using Graphpad Prism 9.0 software. The specific test is described in the figure legend of each figure. A value of $P < 0.05$ was considered statistically significant, where (*) represents $P < 0.05$, (**) represents $P < 0.01$ and (***) represents $P < 0.001$.

## Supplement Figures

### Figure S1

**DMD 7-year-old (7y) male iPSC karyotype, Sanger sequencing and teratoma.** (A) The karyotype results of the 7y iPSCs shows that the cells maintained chromosomal integrity during reprograming. (B, C) Sanger sequencing of both DMD iPSCs and DMD iPSC-CMs, respectively, demonstrated their genetic mutation corresponding to the donor. (D) Teratoma generated from the 7y iPSCs included cell types of the 3 germ layers – endoderm (n), mesoderm (m) and ectoderm (e) (object X15).

### Figure S2

**Waterfall plot displaying the effect size (Cohen's d) for 120 metabolites in the comparisons** (A) DMD adult male vs healthy male; (B) DMD 13-year-old male vs healthy male; (C) DMD 7-year-old male vs healthy male; (D) DMD adult female vs healthy female. Vertical dashed lines indicate threshold for one pooled standard deviation negative difference (in green) and positive difference (in red).

Figure S3

**A diagram depicting the main catabolic pathways contributing to the generation of ATP, including the ATP-to-phosphocreatine balance**. Oxidizable substrates converge through various pathways (glycolysis, beta-oxidation, etc.) in the tricarboxylic acid (TCA) cycle generating ATP and redox cofactors (NADH, $FADH_2$) in several reactions. The main ATP generator is the oxidative phosphorylation (OxPhos) process in the inner mitochondrial membrane, which converts the electrons donated by the redox cofactors in a proton gradient that ultimately is used for the synthesis of ATP from ADP. The adenylate molecules as well as adenosine are a direct read-out for energy status of the cell, as well as phosphocreatine, which can be dephosphorylated to generate ATP in situations of energy crisis by the reverse reaction catalyzed by Creatine kinase (CK).

Figure S4

**Action potential characteristics of spontaneously firing healthy male, DMD adult male, DMD adult female, DMD 7-year-old (7y) and DMD 13-year-old (13y) iPSC-CMs.** (A) Mean beat rate (Beats Per Minute, BPM). (B) Action potential amplitude (APA). (C) Maximal diastolic potential (MDP). (D) Maximal rate of phase 0 depolarization ($dV/dt_{max}$). (E, F) Action potential duration at 20% and 90% of repolarization ($APD_{20}/APD_{90}$). (G-I) Corrected action potential durations: c-$APD_{90}$_B = Bazett-corrected $APD_{90}$=($ADP_{90}$/(Cycle Length)1/2); c$APD_{90}$_H = Hodge's correction: ADP90+1.75 x (heart rate - 60); c$APD_{90}$_Fri (Fridericia's correction: $ADP_{90}$/(Cycle Length)1/3). Healthy male, n = 7; 7y, n = 13; 13y, n=13. The Shapiro–Wilk test of normality was applied for all parameters to assess whether the data were normally distributed. t-test; $^*P < 0.05$, $^{**}P < 0.01$, $^{***}P < 0.001$.

Figure S5

**General explanation of the respirometry analyses following the indicated treatments** (A) as well as the electron transport chain complexes and their specific inhibitors (B).

Figure S6

**Glycolysis in healthy male and DMD iPSC-CMs.** (A-C) Glycolysis represented by extracellular acidification rate (ECAR), measured by XFe96 Seahorse metabolic flux analyzer in 7-year-old (7y, green symbols), adult male (AM, red symbols) and adult female (AM, blue symbols) iPSC-CMs compared to healthy male iPSC-CMs (black symbols). After 1 hr of glucose deprivation, ECAR was measured following glucose injection to delineate basal glycolysis. (D-F) There is no significant change in glycolysis levels in all DMD iPSC-CMs compared to healthy iPSC-CMs. ECAR values are normalized to μg protein, measured using Modified Lowry protein quantitation Assay. Healthy male, n = 7; 7y, n = 12; Adult male, n = 10; Adult female, n = 4. The Shapiro–Wilk test of normality was applied for all parameters to assess whether the data were normally distributed. t-test; NS = non-significant.

Figure S7

**Morphological alterations of DMD iPSC-CM mitochondria.** Transmission electron-microscopy (TEM) analysis of (A) healthy male, (B) 7y male DMD, (C) adult male DMD and (D)

adult female DMD iPSC-CMs. The healthy male and DMD 7y male iPSC-CMs present mitochondria with normal ultrastructure shown in A and B with rare abnormal mitochondria morphology. The adult DMD iPSC-CMs present mitochondrial aberrations, such as increased size (shown in C), reduced matrix density and disrupted cristae (shown in D, orange arrows). S = Sarcomere. Scale bar = 400 nm.

Figure S8

**Negative control of mitochondrial activity staining in healthy male iPSC-CMs.** Representartive confocal microscopy representative mitochondria images in healthy male beating iPSC-CMs simultaneously stained with MTG (green; $\Delta\Psi$ independent), TMRE (red; $\Delta\Psi$ dependent) and Hoechst (blue; Nuclear staining for live cells) for 1 hr, subsequently treated with FCCP or oligomycin (oligo), as a negative control. The Shapiro–Wilk test of normality was applied for all parameters to assess whether the data were normally distributed. t-test; NS = non-significant, $^{**}P < 0.01$, $^{***}P < 0.001$.

## References

1. Eisen B, Jehuda R Ben, Cuttitta AJ, Mekies LN, Shemer Y, Baskin P, Reiter I, Willi L, Freimark D, Gherghiceanu M, Monserrat L, Scherr M, Hilfiker-Kleiner D, Arad M, Michele DE, Binah O. Electrophysiological abnormalities in induced pluripotent stem cell-derived cardiomyocytes generated from Duchenne muscular dystrophy patients. *J Cell Mol Med* 2019;**23**:2125–2135.
2. Eisen B, Jehuda R Ben, Cuttitta AJ, Mekies LN, Reiter I, Ramchandren S, Arad M, Michele DE, Binah O. Generation of Duchenne muscular dystrophy patient-specific induced pluripotent stem cell line lacking exons 45–50 of the dystrophin gene (IITi001-A). *Stem Cell Res* 2018;**29**:111–114.
3. Jimenez-Vazquez EN, Arad M, Macías Á, Vera-Pedrosa L, Cruz-Uréndez FM, Cuttitta AJ, Monteiro Da Rocha A, Herron TJ, Ponce-Balbuena D, Guerrero-Serna G, Binah O, Michele DE. SNTA1 Gene rescues ion channel function in cardiomyocytes derived from induced Pluripotent Stem Cells reprogrammed from Muscular Dystrophy patients with arrhythmias. *Elife* 2022;**11**:1–30.
4. Ben Jehuda R, Eisen B, Shemer Y, Mekies LN, Szantai A, Reiter I, Cui H, Guan K, Haron-Khun S, Freimark D, Sperling SR, Gherghiceanu M, Arad M, Binah O. CRISPR correction of the *PRKAG2* gene mutation in the patient's iPSC-derived cardiomyocytes eliminates the electrophysiological and structural abnormalities. *Heart Rhythm* 2017;1–10.
5. Yehezkel S, Rebibo-Sabbah A, Segev Y, Tzukerman M, Shaked R, Huber I, Gepstein L, Skorecki K, Selig S. Reprogramming of telomeric regions during the generation of human induced pluripotent stem cells and subsequent differentiation into fibroblast-like derivatives. *Epigenetics* 2011;**6**:63–75.
6. Novak A, Barad L, Lorber A, Gherghiceanu M, Reiter I, Eisen B, Eldor L, Itskovitz-Eldor J, Eldar M, Arad M, Binah O. Functional abnormalities in iPSC-derived cardiomyocytes generated from CPVT1 and

CPVT2 patients carrying ryanodine or calsequestrin mutations. *J Cell Mol Med* 2015;**19**:2006–2018.

7. Streckfuss-Bömeke K, Wolf F, Azizian A, Stauske M, Tiburcy M, Wagner S, Hübscher D, Dressel R, Chen S, Jende J, Wulf G, Lorenz V, Schön MP, Maier LS, Zimmermann WH, Hasenfuss G, Guan K. Comparative study of human-induced pluripotent stem cells derived from bone marrow cells, hair keratinocytes, and skin fibroblasts. *Eur Heart J* 2013;**34**:2618–2629.
8. Novak A, Barad L, Zeevi-Levin N, Shick R, Shtrichman R, Lorber A, Itskovitz-Eldor J, Binah O. Cardiomyocytes generated from CPVT D307H patients are arrhythmogenic in response to betha-adrenergic stimulation. *J Cell Mol Med* 2012;**16**:468–482.
9. MacKay GM, Zheng L, Broek NJF Van Den, Gottlieb E. Analysis of cell metabolism using LC-MS and isotope tracers. *Methods in Enzymology* 2015. p. 171–196.
10. Anders S, Huber W. Differential expression analysis for sequence count data. *Genome Biol* BioMed Central; 2010;**11**:1–12.
11. Pietzke M, Vazquez A. Metabolite AutoPlotter - an application to process and visualise metabolite data in the web browser. *Cancer Metab 2020 81* BioMed Central; 2020;**8**:1–11.
12. Ben-Ari M, Schick R, Barad L, Novak A, Ben-Ari E, Lorber A, Itskovitz-Eldor J, Rosen MR, Weissman A, Binah O. From beat rate variability in induced pluripotent stem cell-derived pacemaker cells to heart rate variability in human subjects. *Heart Rhythm* 2014;**11**:1808–1818.
13. Mandel Y, Weissman A, Schick R, Barad L, Novak A, Meiry G, Goldberg S, Lorber A, Rosen MR, Itskovitz-Eldor J, Binah O. Human embryonic and induced pluripotent stem cell-derived cardiomyocytes exhibit beat rate variability and power-law behavior. *Circulation* 2012;**125**:883–893.
