# Supplementary figures and images for: Bioenergetic and Metabolic Impairments in Induced Pluripotent Stem Cell-Derived Cardiomyocytes Generated from Duchenne Muscular Dystrophy Patients
Source: Int J Mol Sci. 2022 Aug 29;23(17):9808. doi: 10.3390/ijms23179808 (PMC9456153; doi:10.3390/ijms23179808)

Figure S1

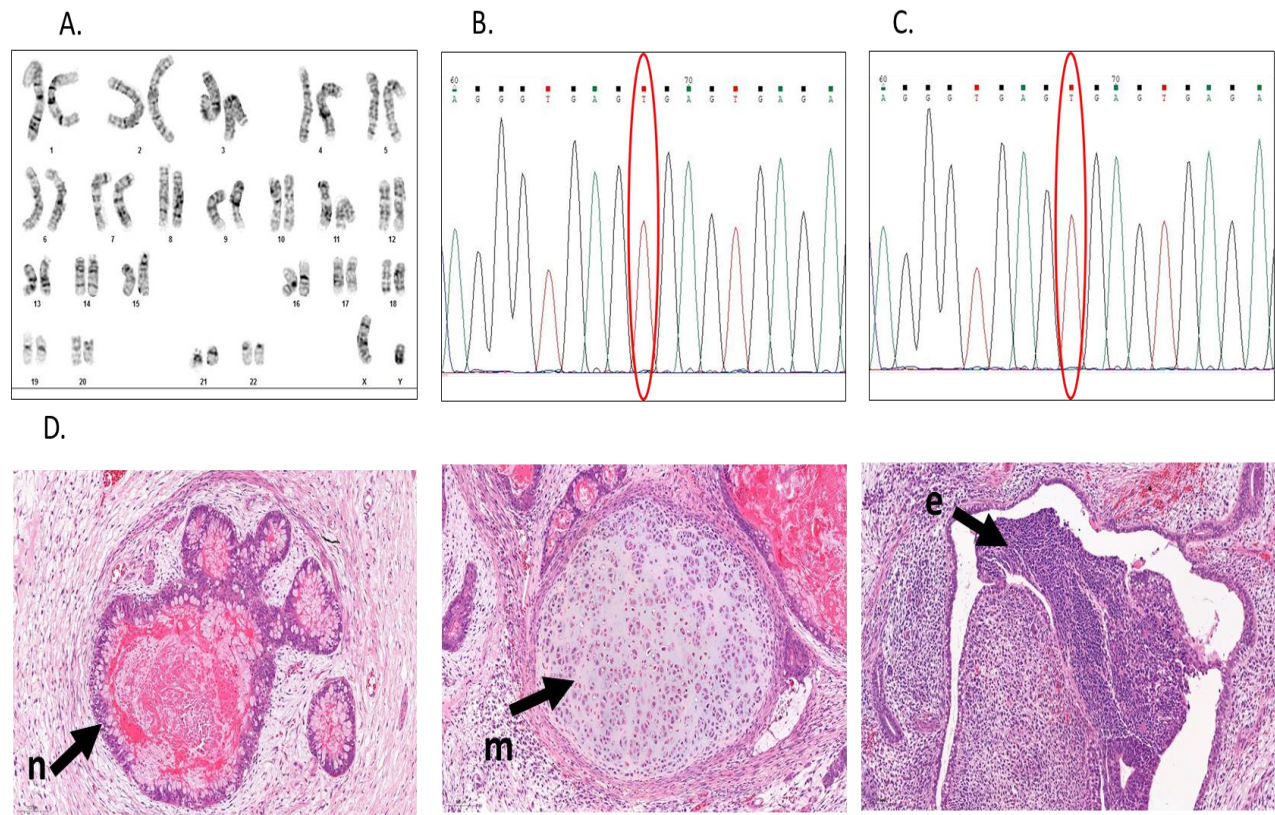

Supplement: Supplementary file 1 [file ijms-23-09808-s001.zip › Figure S1.pdf]

Figure S2

A DMD Adult male vs healthy male

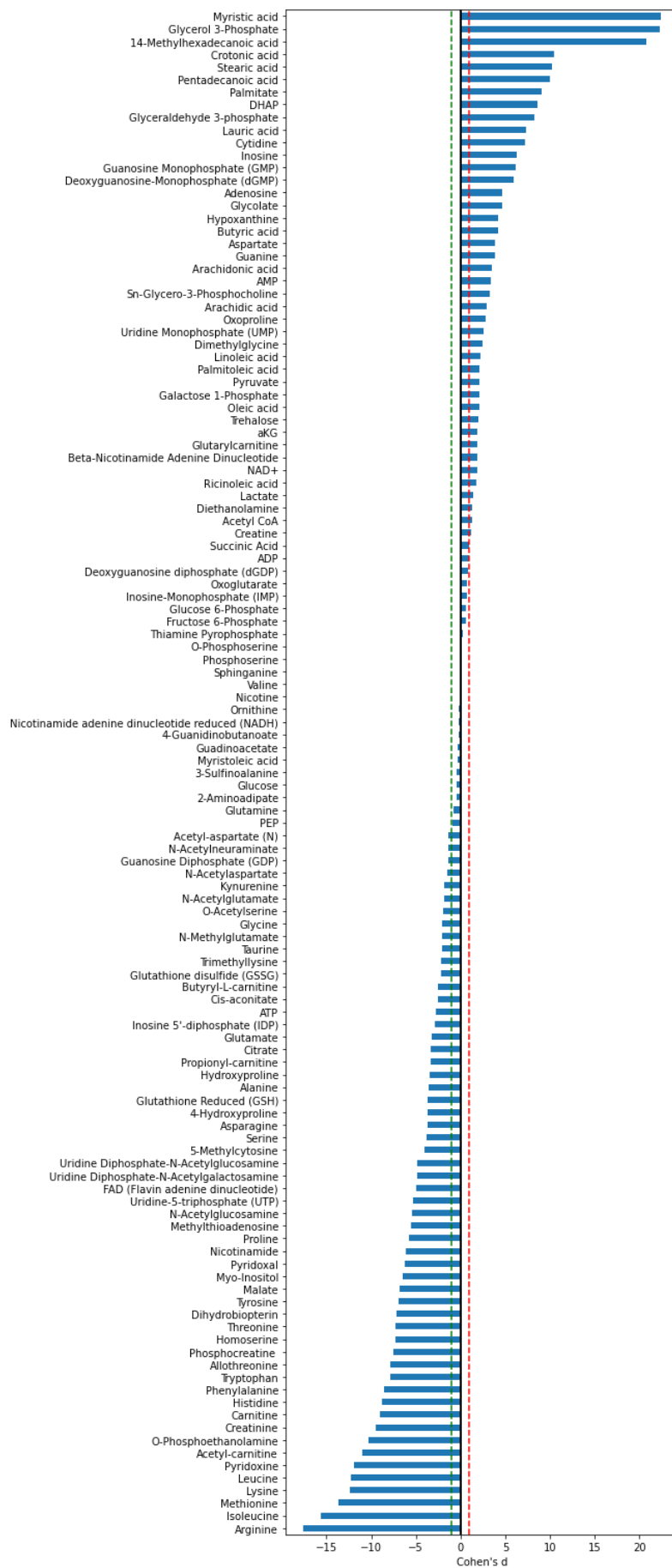

Supplement: Supplementary file 1 [file ijms-23-09808-s001.zip › Figure S2A.pdf]

Figure S2-cont.

B DMD 13-years old male vs healthy male

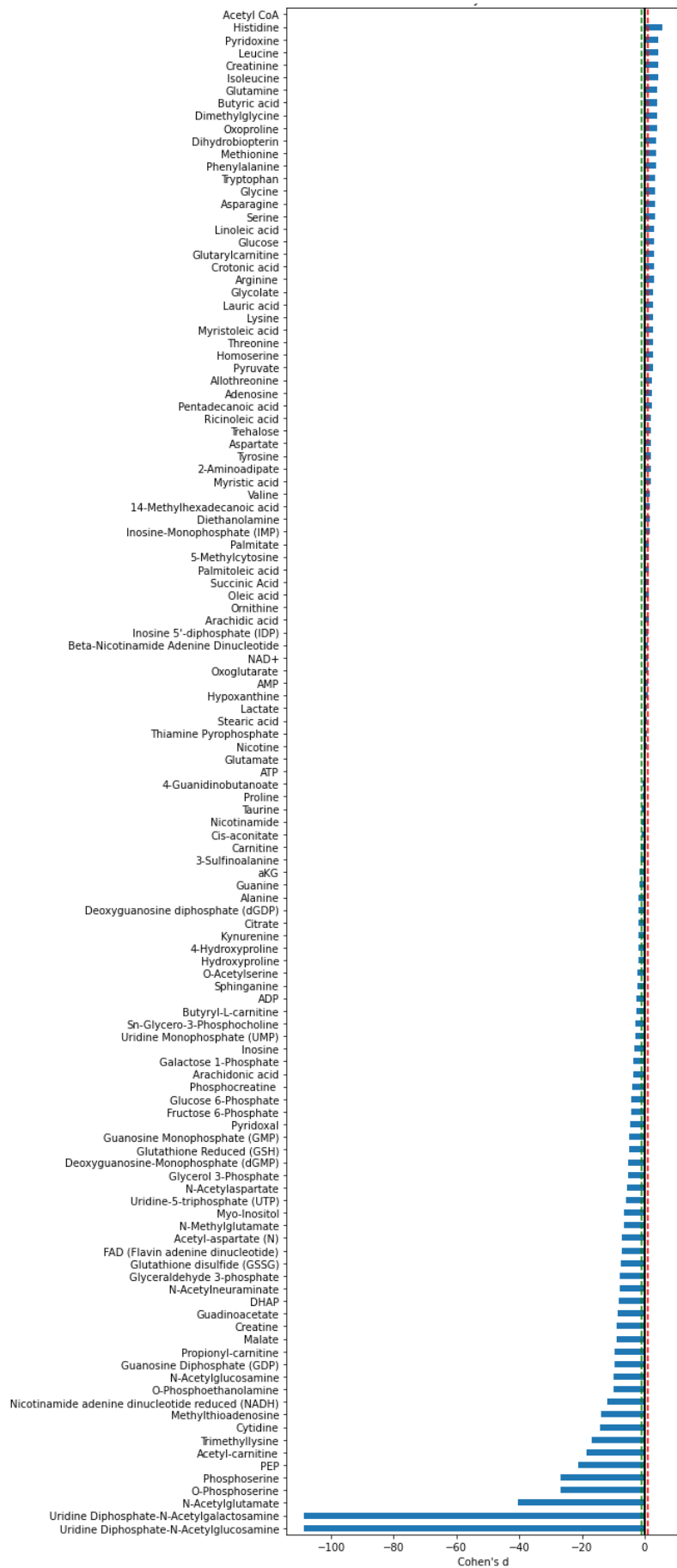

Supplement: Supplementary file 1 [file ijms-23-09808-s001.zip › Figure S2B.pdf]

Figure S2-cont.

c DMD 7-years old male vs healthy male

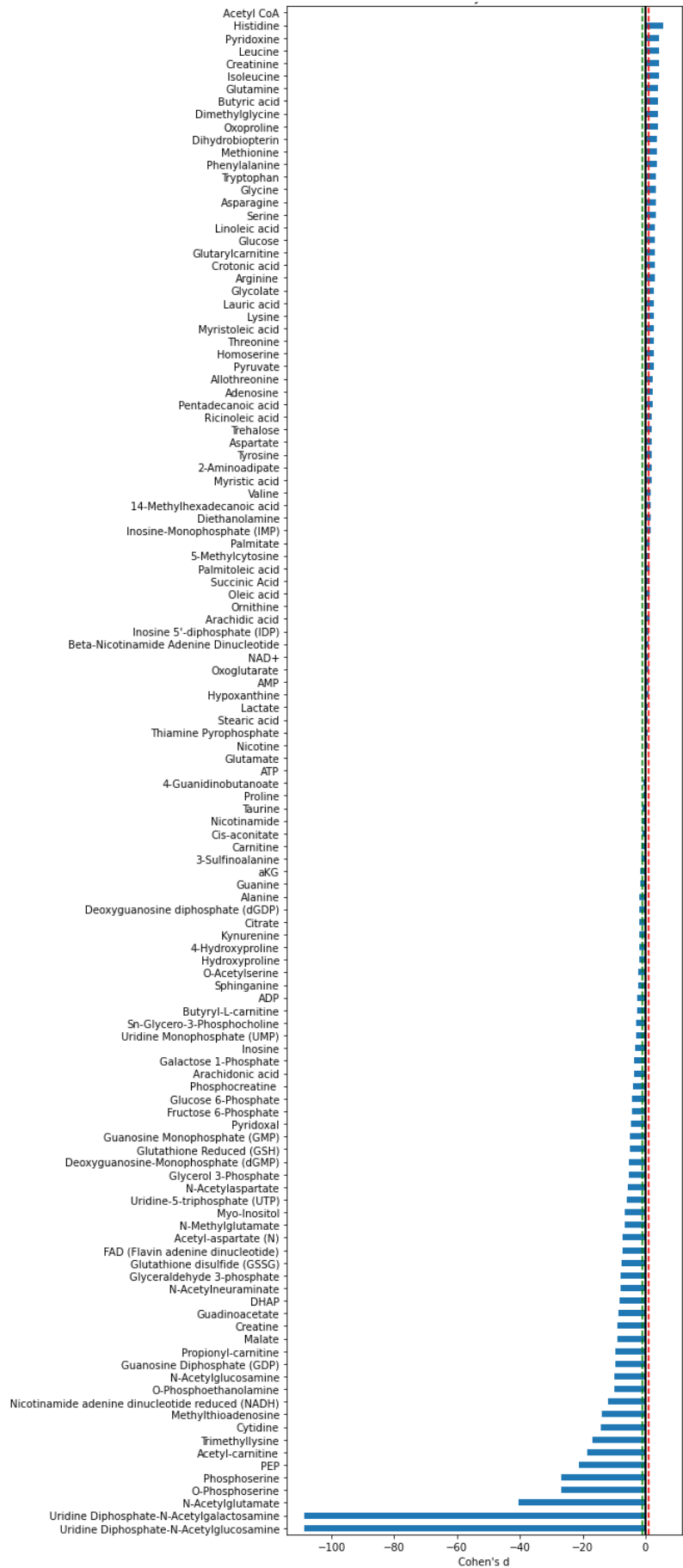

Supplement: Supplementary file 1 [file ijms-23-09808-s001.zip › Figure S2C.pdf]

Figure S2-cont.

D DMD Adult female vs healthy female

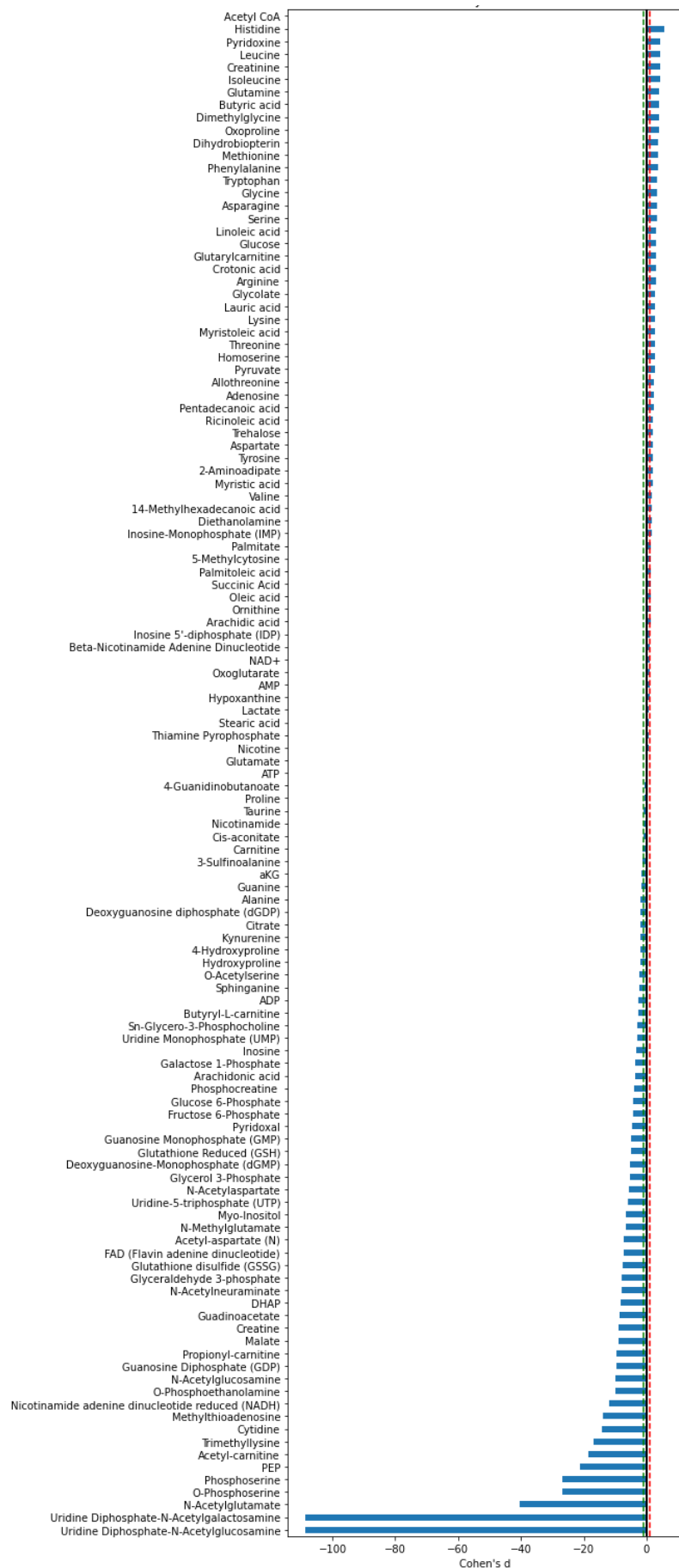

Supplement: Supplementary file 1 [file ijms-23-09808-s001.zip › Figure S2D.pdf]

Figure S3

Fatty acids

Cytosol

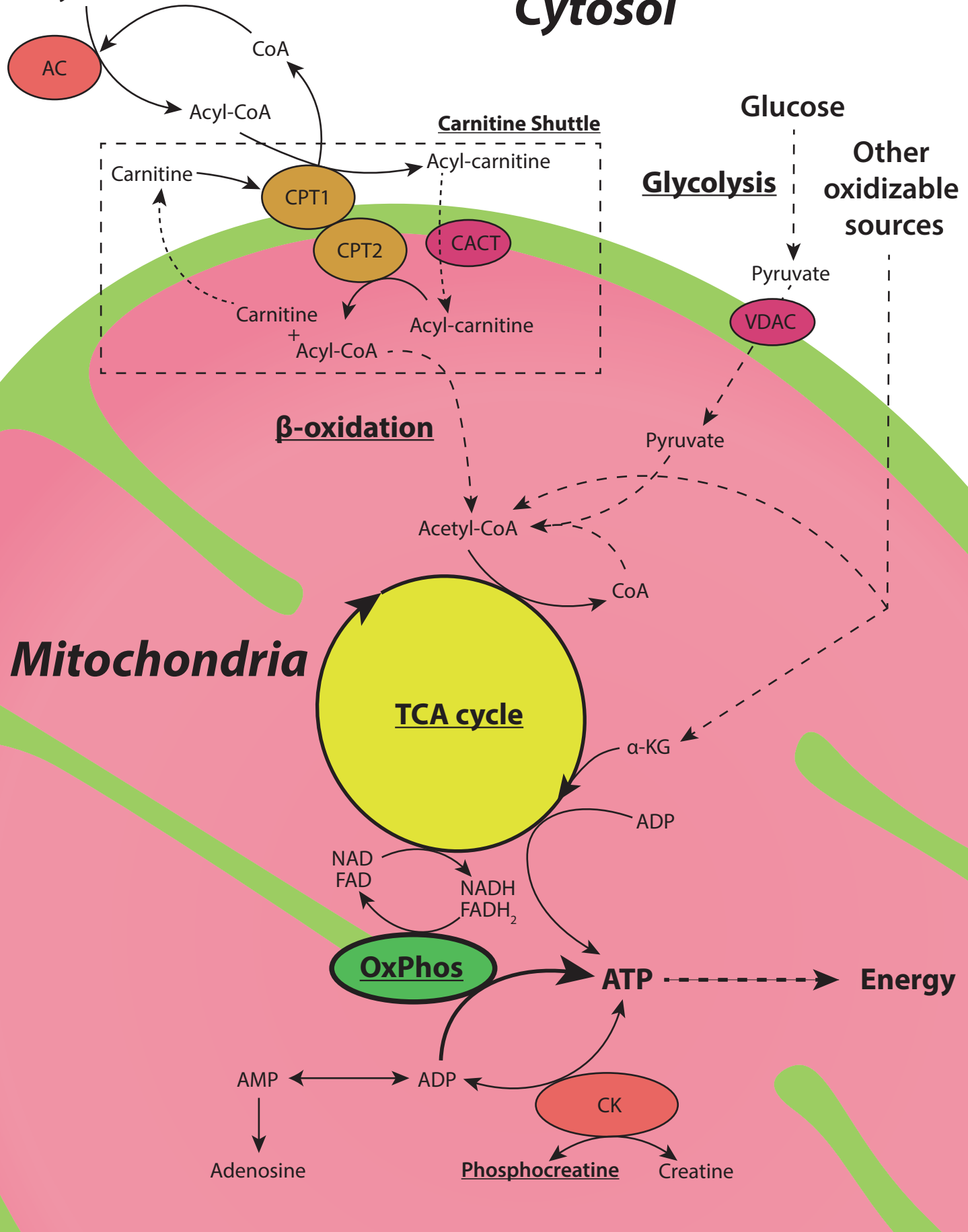

Supplement: Supplementary file 1 [file ijms-23-09808-s001.zip › Figure S3.pdf]

**Figure S4**

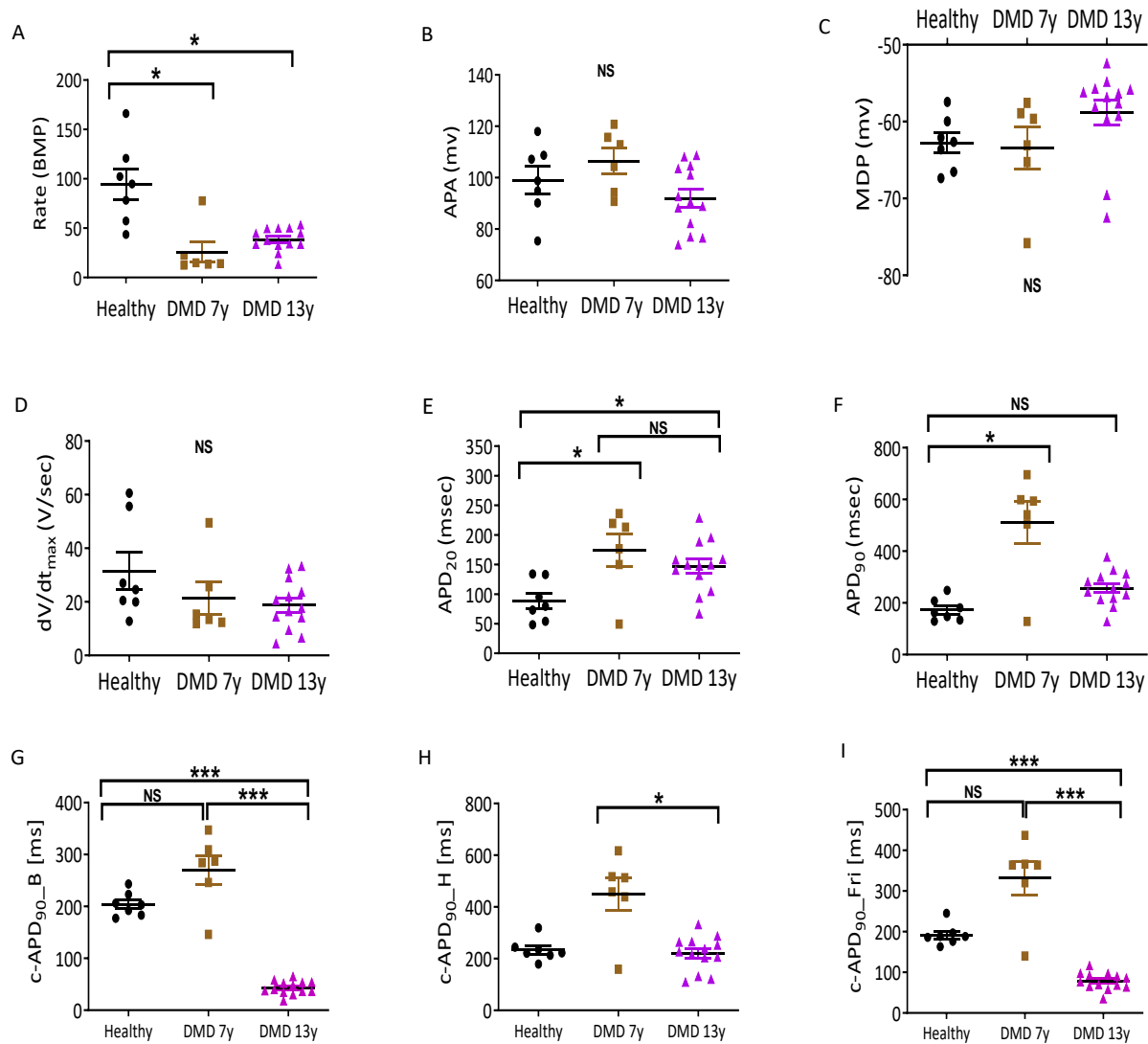

Supplement: Supplementary file 1 [file ijms-23-09808-s001.zip › Figure S4.pdf]

A

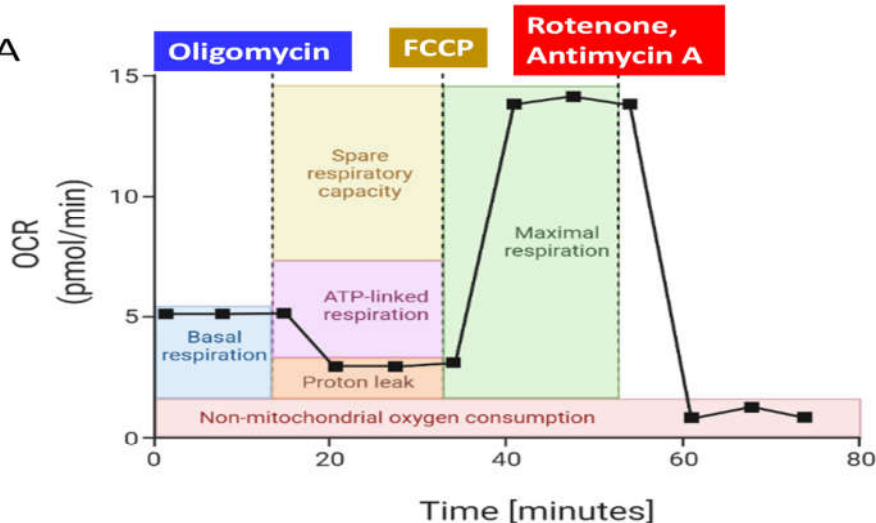

B

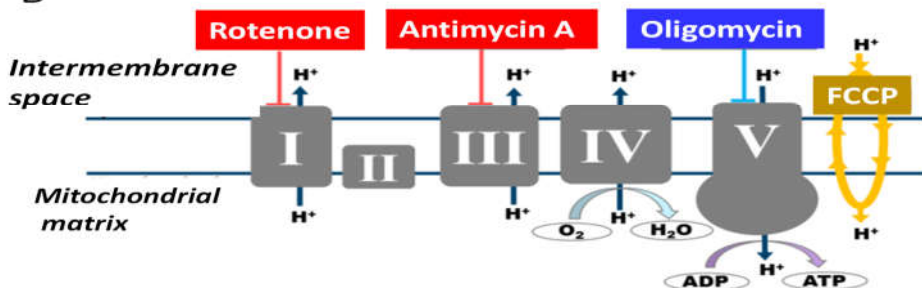

Supplement: Supplementary file 1 [file ijms-23-09808-s001.zip › Figure S5.pdf]

A

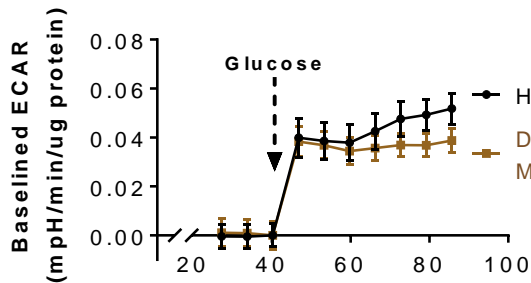

B

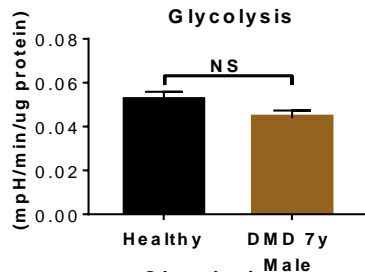

C

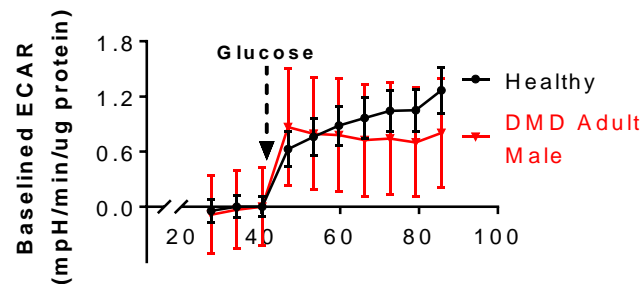

D

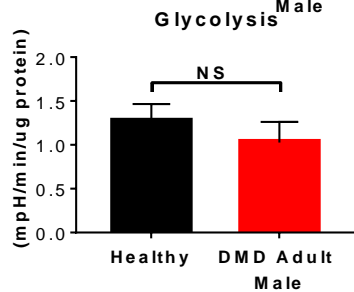

E

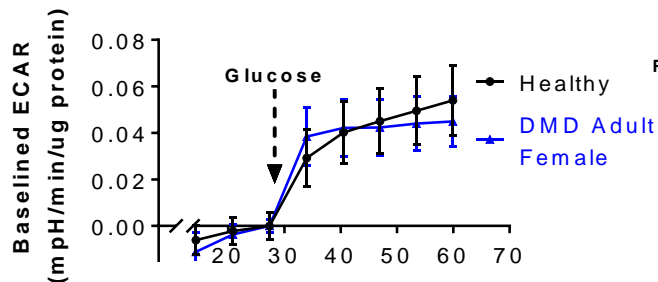

F

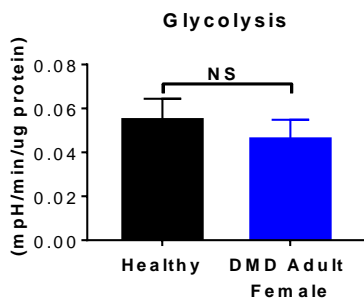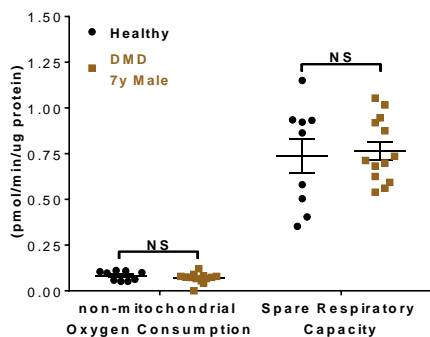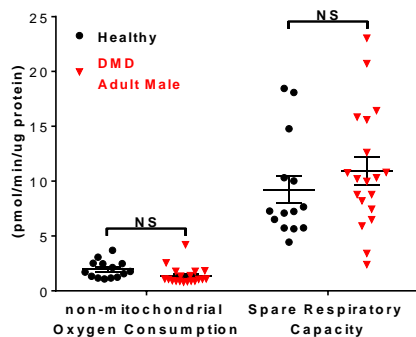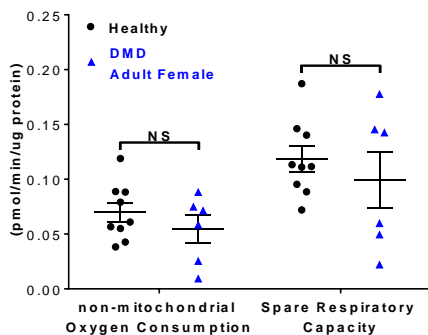

Supplement: Supplementary file 1 [file ijms-23-09808-s001.zip › Figure S6.pdf]

**A****Healthy Male**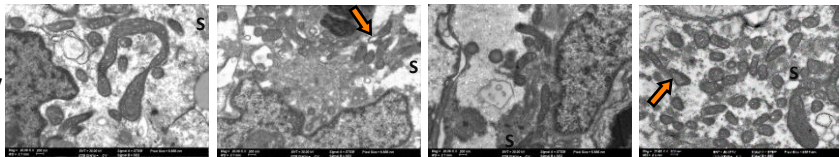**B****DMD 7y Male**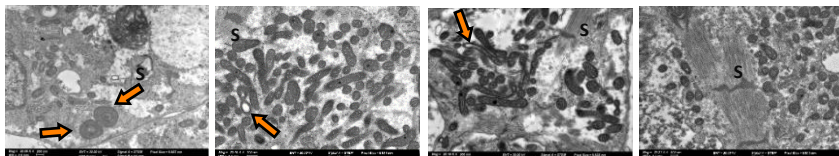**C****DMD Adult male**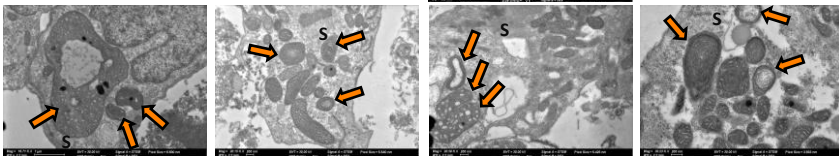**D****DMD Adult female**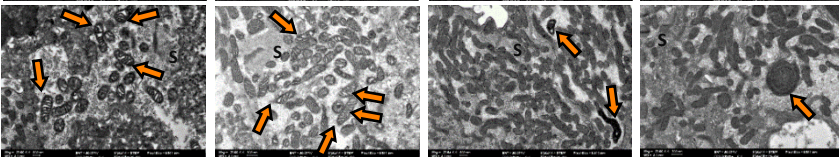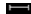**400nm**

Supplement: Supplementary file 1 [file ijms-23-09808-s001.zip › Figure S7.pdf]

**A****MTG****TMRE****Merge****Healthy**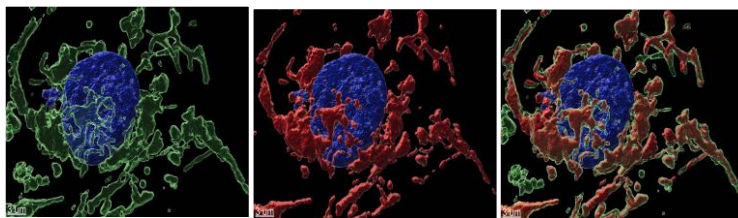**Healthy  
+ FCCP**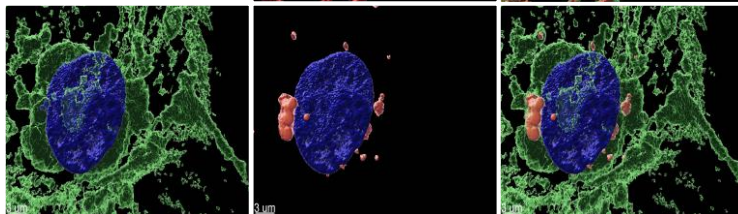**Healthy**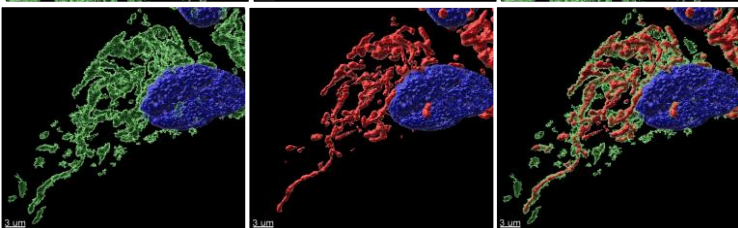**Healthy  
+ Oligo**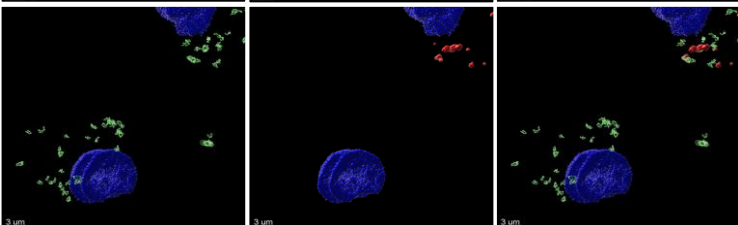**Mitochondrial Activity**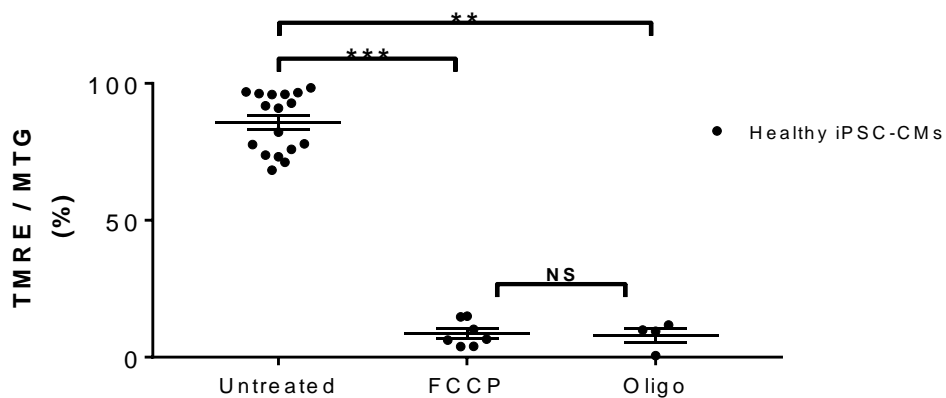

Supplement: Supplementary file 1 [file ijms-23-09808-s001.zip › Figure S8.pdf]
